# Climate Warming May Increase the Transboundary Expansion Risk of *Calliptamus italicus* Between Xinjiang, China and Kazakhstan

**DOI:** 10.3390/insects17070710

**Published:** 2026-07-09

**Authors:** Manfei Bai, Jianghua Zheng, Jun Lin, Zhong Liang, Feifei Zhang, Junteng Luo, Xiaoyu Guo, Xuan Li, Ke Zhang, Jianguo Wu, Qi Qige

**Affiliations:** 1 College of Geography and Remote Sensing Sciences, Xinjiang University, Urumqi 830046, China; 107552401152@stu.xju.edu.cn (M.B.); zhangfeifei@stu.xju.edu.cn (F.Z.); 107556524203@stu.xju.edu.cn (J.L.); 107552301174@stu.xju.edu.cn (X.G.); 107556523284@stu.xju.edu.cn (X.L.); 107552303793@stu.xju.edu.cn (K.Z.); 2 Xinjiang Key Laboratory of Oasis Ecology, Xinjiang University, Urumqi 830046, China; 3 Center for Grassland Biological Disaster Prevention and Control of Xinjiang, Urumqi 830000, China; xjcy2009@163.com (J.L.); 18999280970@163.com (Z.L.); zhbshuju@163.com (J.W.); enkenm@163.com (Q.Q.)

**Keywords:** *Calliptamus italicus*, climate change, species distribution models, transboundary spread risk, niche dynamics

## Abstract

The Italian locust (*Calliptamus italicus*) is a major agricultural pest causing significant damage to crops and grasslands in arid and semi-arid regions. To investigate its response to climate change, we applied ensemble species distribution modeling combined with niche analysis to predict its current and future potential distributions, and assess climatic niche dynamics and potential transboundary spread risks. We found that key environmental drivers included altitude, temperature variables (bio10, bio9), precipitation variables (bio18, bio17), and human footprint. Suitable habitat of *Calliptamus italicus* currently accounts for approximately 9.65% of the study area; under various future climate scenarios, this proportion is projected to rise to between 10.12% and 11.93%. Niche dynamics analysis revealed strong climatic niche conservatism and consistently high niche overlap across scenarios, suggesting that future range expansion is more likely to occur through tracking climatically analogous habitats rather than niche shifts. These findings provide spatially explicit support for risk zoning, monitoring prioritization, and the development of early-warning and adaptive management strategies, particularly within a China–Kazakhstan transboundary monitoring and biosecurity framework.

## Simple Summary

The Italian locust is a major agricultural pest that threatens pastures and crops in the arid and semi-arid regions of Central Asia and northwestern China. Climate change is likely to alter its distribution range and increase the risk of cross-border spread. This study integrates species distribution modeling with niche analysis to predict changes in the species’ distribution across Xinjiang, China, and Kazakhstan under different climate scenarios. The results indicate that temperature, precipitation, topography, and human activities are the primary factors influencing its distribution. Climate warming will drive the expansion of suitable habitats, potentially increasing the risk of cross-border spread. The species’ climatic niche remains generally stable across different scenarios, suggesting that population expansion tends to occur through migration to climatically suitable regions rather than adaptation to entirely new environments. The findings of this study can serve as a reference for cross-border monitoring, disaster early warning, and long-term pest control in the context of climate change.

## 1. Introduction

The Italian locust, *Calliptamus italicus* (L.) (*Orthoptera: Acridoidea*), is a polyphagous grasshopper species [1,2,3]. Its distribution ranges from Southern Europe to the Altai Mountains (including Central Russia, Turkey, and NW China), this species frequents arid steppes, deserts, and Mediterranean drylands [4,5]. *Calliptamus italicus* exhibits strong environmental adaptability and a high outbreak potential. Under favorable climatic conditions, it can form high-density populations, causing severe damage to agricultural crops and natural grasslands, and is therefore considered a major economic pest of grasslands, cotton, cereals, and alfalfa across Central Asia [1,3]. Moreover, its pest risk is not only reflected in the rapid increase of local population densities but is also closely associated with its strong migratory and dispersal capacity. As a typical migratory pest, *Calliptamus italicus* exhibits strong flight capacity in its gregarious adult stage, enabling medium-distance migrations over relatively short periods (typically ~100–200 km), thereby facilitating the spread and expansion of outbreaks at the regional scale [3,6,7,8].Although nymphal bands also possess migratory capacity, they tend to remain within the same habitat for several weeks under conditions of sufficient food resources, exhibiting pronounced local aggregation patterns [5,9]. During the 20th century, large-scale outbreaks of *Calliptamus italicus* were mainly concentrated in three specific decades: 1921–1930, 1931–1940, and 1991–2000 [7,10]. Among these periods, the outbreaks at the end of the last century were particularly severe, with more than 16.6 million hectares affected in Kazakhstan and Russia. During this period, Kazakhstan alone spent over USD 23 million on locust control campaigns [7]. As Xinjiang, China, and Kazakhstan are located within the core distribution area of locusts in Central Asia, sharing similar ecological environments and contiguous borders, this geopolitical and ecological setting substantially increases the risk of transboundary locust spread. Statistics indicate that from 1980 to 2011, the average annual locust-affected area along the China–Kazakhstan border reached approximately 3300 km^2^ [11], with individual outbreak events causing economic losses of about CNY 14 million [12]. In the face of such persistent and severe biological hazards, strengthening the monitoring and control of *C. italicus* outbreaks is not only critical for regional pest management but also represents a key measure for safeguarding food security and ecological stability at both regional and broader scales.

Global temperatures have increased by approximately 1.1 °C above pre-industrial levels due to human activities, resulting in increasingly irreversible impacts on ecosystems [13]. In response to mounting environmental pressures, species commonly shift their distributions toward higher latitudes or elevations to track suitable habitats, and such geographical redistribution has become a widespread global phenomenon [6,14]. As a typical migratory pest, *Calliptamus italicus* is particularly sensitive to climate change. Its strong flight capacity and high environmental adaptability may facilitate redistribution into newly suitable areas and increase the likelihood of transboundary spread across Central Asia, and posing new challenges to traditional pest management systems that are largely based on administrative boundaries [6,9]. Meanwhile, the increasing aridification associated with global warming closely matches the xerophilic biological traits of *C. italicus*, potentially promoting large-scale outbreaks of this species [15,16].

Species distribution models (SDMs) are widely used to evaluate species environment relationships and predict potential distribution shifts under climate change [17]. However, predictions from single-algorithm models may be inconsistent, leading to increased uncertainty, particularly under future climate scenarios [18]. To reduce model uncertainty, we used the Biomod2 ensemble framework (biomod2: Ensemble Platform for Species Distribution Modeling), which combines multiple algorithms to enhance predictive accuracy and robustness [18]. Biomod2 has been widely applied in species distribution modeling, particularly in studies of insect distribution and pest risk assessment [19].

Previous studies in Central Asia have explored the impacts of climate change on the distribution patterns of multiple grassland locust species [20,21,22]. For example, Zhang et al. (2026) evaluated the future habitat changes of two migratory locust species along the China–Kazakhstan border under climate change scenarios, with a particular focus on the influence of environmental conditions during different developmental stages on locust distribution [23]. However, these studies have mainly focused on habitat changes or outbreak trends across multiple locust species, with limited attention given to the species-specific climatic responses and transboundary distribution mechanisms of *Calliptamus italicus*. In particular, few studies have explored whether the future expansion of *C. italicus* is likely to occur through climatic niche shifts or through the tracking of climatically analogous habitats.

Accordingly, this study employs the Biomod2 platform to develop an ensemble species distribution model for *C. italicus* by integrating multiple environmental variables and climate change scenarios. We project habitat suitability for *C. italicus* in Xinjiang, China and Kazakhstan under present and future climate conditions and assess the potential risk of transboundary spread. The study aims to (i) identify the key environmental drivers of species distribution, (ii) analyze changes in habitat suitability across space and time, and (iii) assess climatic niche dynamics in response to climate change. These findings enhance understanding of species responses to changing environments and provide an empirical foundation for designing adaptive, cross-border monitoring networks and early-warning systems.

## 2. Materials and Methods

### 2.1. Acquisition and Pre-Processing of Calliptamus Italicus Occurrence Records

A total of 1067 occurrence records of *Calliptamus italicus* were compiled for constructing an accurate species distribution model. Part of the data was obtained from the Global Biodiversity Information Facility (GBIF Occurrence Download, Available online: https://doi.org/10.15468/dL.4rwzhz, accessed on 25 November 2025) and published literature [24]. Occurrence records were further enriched through field investigations carried out across Xinjiang, China between 2020 and 2024.

**Figure 1:**
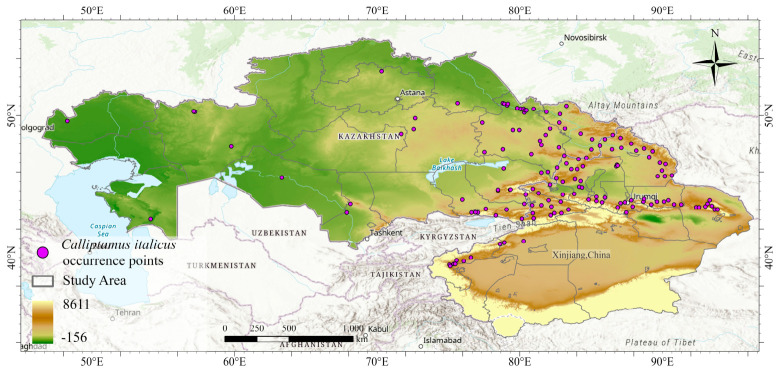
Distribution points of the *Calliptamus italicus*.

To ensure data accuracy and reliability, records with incomplete geographic coordinates or duplicate locations were removed. Spatial autocorrelation among occurrence points was further reduced using ENMTools by filtering redundant records within the same raster cell. To align with the 5 km × 5 km resolution of the environmental variables, a maximum of one occurrence record was retained per grid cell. After data cleaning and spatial thinning, a total of 559 valid occurrence records of *C. italicus* were retained within the study area (Figure 1). The final occurrence dataset was converted into CSV format for subsequent modeling analyses.

### 2.2. Screening Environmental Variables

When selecting environmental variables for analysis, four categories of factors were considered based on the biological and ecological characteristics of *Calliptamus italicus*: bioclimatic, soil, topographic, and anthropogenic factors. Environmental variables were selected based on the life-history traits and ecological requirements of *C. italicus*. Bioclimatic variables influence survival, development, and reproduction; soil properties affect oviposition and egg overwintering; topographic factors shape local microclimatic conditions; and anthropogenic factors may alter habitat availability and disturbance regimes [22,25,26,27,28]. When selecting environmental variables for analysis, four categories of factors were considered based on the biological and ecological characteristics of *Calliptamus italicus*: bioclimatic, soil, topographic, and anthropogenic factors (Table S5). Bioclimatic variables were obtained from the WorldClim version 2.1 database (https://www.worldclim.org/, accessed on 2 October 2025). Monthly historical climate data for the period 2001–2020 were assembled with a spatial resolution of 2.5 arc-minutes, consisting of total precipitation (mm), mean minimum temperature (°C), and mean maximum temperature (°C). Nineteen standard bioclimatic variables characterizing recent climate conditions were generated with the biovars function implemented in the R package dismo. Topographic variables included elevation, slope, and aspect [29]. Global elevation data were obtained from the WorldClim v2.1 database (https://www.worldclim.org/, accessed on 2 October 2025) at a spatial resolution of 2.5 arc-minutes. Slope and aspect were calculated from elevation data using ArcGIS10.2. Soil variables were obtained from the SoilGrids250m v2.0 dataset (https://isric.org/explore/soilgrids/, accessed on 2 October 2025). In this study, 11 soil properties related to soil physicochemical characteristics were selected at a depth of 0–5 cm at 250-m spatial resolution. Human disturbance was represented by the Global Human Footprint dataset, provided by the Urban Environment Monitoring and Modeling team at China Agricultural University, with a resolution of 1 km [30]. The dataset comprises eight indicators reflecting various aspects of human pressure, such as built-up areas, population density, nighttime lights, cropland, pasture, roads, railways, and navigable waterways. Following the approach of Sanderson and Venter et al., annual global human footprint maps were generated for the period 2000–2020.

Climate projections for the study were derived from the BCC-CSM2-MR model (CMIP6) for a combination of three SSPs (SSP1-2.6, SSP2-4.5, SSP5-8.5) and two time periods (2030s and 2050s), resulting in six future scenarios (https://www.worldclim.org/, accessed on 2 October 2025) [24,31,32,33].

To meet the requirements of the multi-model framework in Biomod2 for consistent environmental data formats, all environmental variables were resampled to a uniform spatial resolution of 0.05° using ArcGIS, with the geographic coordinate system set to GCS_WGS_1984.

**Figure 2:**
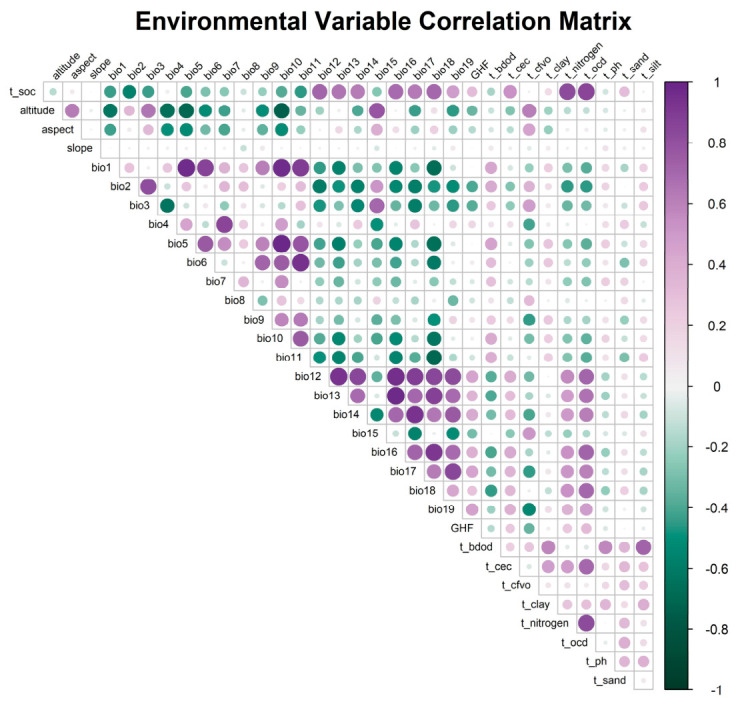
Pearson correlation analysis of the environmental variables.

Environmental variables are critical inputs for ecological niche modeling; however, strong correlations among variables can lead to model overfitting and reduced predictive accuracy [34]. The initial set of 33 environmental variables underwent Pearson correlation analysis to identify and remove highly correlated variables, thereby enhancing model reliability (Figure 2). Highly correlated environmental factors (|r| ≥ 0.75) were excluded, and the MaxEnt jackknife procedure was used as a preliminary variable-screening approach to identify predictors with low explanatory contribution to *C. italicus* habitat suitability. Ultimately, 19 environmental variables were retained for subsequent model construction, as listed in Table S1.

### 2.3. Model Construction and Evaluation

The analysis employed the Biomod2 package (v4.2-6-2) in R to construct an ensemble modeling framework, leveraging its integrated algorithms for species distribution modeling [18]. Initially, 12 algorithms were considered. However, the Artificial Neural Network (ANN) algorithm repeatedly returned a failed model status during model calibration in Biomod2 and therefore did not generate valid outputs for subsequent evaluation. Consequently, ANN was excluded from the final analyses, and the remaining 11 algorithms were retained for ensemble modeling. Therefore, ANN was excluded from the final analyses, and the remaining 11 algorithms were used for ensemble modeling. The algorithms successfully implemented and retained for modeling included: Generalized Linear Model (GLM), Generalized Boosting Model (GBM), Generalized Additive Model (GAM), Classification Tree Analysis (CTA), Flexible Discriminant Analysis (FDA), Multivariate Adaptive Regression Splines (MARS), Random Forest (RF), Maximum Entropy Model (MaxEnt), Maximum Entropy Model with Lasso Regularization (MaxNet), Extreme Gradient Boosting (XGBoost), and Surface Range Envelope (SRE). The Biomod2 framework requires both presence and absence data for model calibration. As true absence data for *Calliptamus italicus* were unavailable, pseudo-absence data were generated to better approximate the species’ actual distribution and to reduce the effects of spatial sampling bias. The Biomod2 package facilitated the generation of five independent pseudo-absence datasets. Each dataset comprised 2500 points randomly selected from background areas with no recorded presences (Figure S5). These pseudo-absence points were used as substitutes for true absence data in model construction.

The Xinjiang, China–Kazakhstan region was defined as the calibration area because it represents the major contemporary distribution and potential dispersal region of *C. italicus* in Central Asia. Environmental variables were clipped to this region, and pseudo-absence points were generated within the same extent. Therefore, model calibration was conducted using the environmental conditions available across the entire study area rather than only within the spatial extent occupied by occurrence records.

All modeling datasets, including presence and pseudo-absence points, were randomly divided into training (75%) and testing (25%) subsets. During both calibration and evaluation, presence and pseudo-absence samples were assigned equal weights.

Two commonly used metrics, the receiver operating characteristic (AUC) curve and the true skill statistic (TSS), were employed to assess model performance [35]. AUC values lie between 0 and 1, with scores above 0.9 denoting high model accuracy. TSS ranges from −1 to 1, where values over 0.75 suggest robust predictive performance [36,37,38]. The combined use of AUC and TSS provides a comprehensive assessment of model accuracy and its agreement with observed species distributions.

To enhance predictive reliability, a stringent model selection criterion was applied. Only models with TSS > 0.75 and AUC > 0.9 were retained for subsequent ensemble forecasting [18,39,40]. Ensemble predictions were generated using four ensemble techniques implemented in the Biomod2 framework: ensemble model concomitant approach (EMca), ensemble model weighted mean (EMwmean), ensemble model mean (EMmean), and ensemble model median (EMmedian). The ensemble outputs were merged to produce an optimal continuous probability prediction, which was then used to assess habitat suitability dynamics under current and future climate conditions [18].

The ensemble model generated continuous species occurrence probability values ranging from 0 to 1000. Based on the AUC-optimized threshold (cutoff = 473.5; sensitivity = 92.251%; specificity = 90.337%), habitats were classified as unsuitable (≤473.5) and suitable (>473.5). For visualization and comparative analyses, suitable habitats were further divided into low-, medium-, and high-suitability classes using the equal-interval classification method. These classes represent relative gradients of habitat suitability and are intended for map interpretation only, rather than biologically meaningful ecological threshold [41,42]. In addition, to identify the environmental conditions associated with highly suitable habitats, response curves were interpreted using a species occurrence probability threshold of 0.6 [42]. When the probability of occurrence of *Calliptamus italicus* was ≥0.6, the response curves revealed clear relationships between habitat suitability and key environmental variables.

The area of suitable habitat was estimated by counting the number of raster cells classified as suitable and multiplying by the area represented by each grid cell. Area calculations were performed in ArcGIS based on the projected raster layers derived from the ensemble model outputs. The total areas of low-, medium-, and high-suitability habitats were then summed to obtain the overall suitable habitat area.

### 2.4. Ecological Niche Dynamics of Calliptamus italicus

Based on the ensemble model predictions, the ecological niche breadth of *Calliptamus italicus* was quantified using the ENMTools package by calculating Levins’ B1 (inverse concentration) and B2 (uncertainty) indices for each time period [43,44]. Both B1 and B2 range from 0 to 1, with higher B1 values indicating a more homogeneous distribution and a broader ecological niche, whereas lower B1 values indicate a more aggregated distribution and a narrower niche [45,46].

Climatic niche dynamics were further quantified using the ecospat package in R v4.5.2. Environmental space was constructed through principal component analysis (PCA), using environmental variables extracted from the entire Xinjiang, China–Kazakhstan region, which was defined as the accessible area for *C. italicus*. Environmental conditions associated with model-predicted suitable habitats under current and future climate scenarios were subsequently projected onto the first two PCA axes to characterize climatic niche occupancy within the environmental space. Kernel density smoothing was applied to estimate species density in environmental space, and a 75% kernel-density threshold was used to reduce the influence of marginal environmental conditions and emphasize the core climatic niche occupied by the species. Therefore, niche comparisons were conducted within the PCA-defined environmental space while niche occupancy was estimated from environmental conditions associated with suitable habitats predicted by the ensemble model.

Schoener’s D and the Hellinger-based I index were used to evaluate niche and range overlap, respectively. We quantified three major components of climatic niche dynamics—stability, expansion, and unfilling—by comparing current and future climatic conditions [47]. Component proportions range from 0% to 100%, with values above 10% typically considered significant. Both D and I values fall between 0 and 1, where values approaching 1 reflect higher degrees of niche and range overlap [47].

In addition, niche equivalency and similarity analyses were conducted. The equivalency test was conducted by generating null distributions through 100 randomizations to compare the observed overlap index (D) with the randomized expectation (D1), thereby assessing whether the niches were statistically equivalent [48]. The similarity test likewise generated a null distribution (D2) to evaluate whether the observed niche overlap was significantly greater than expected by chance, indicating structural similarity between niches. Statistical significance was evaluated using a 95% confidence interval, with *p* < 0.05 indicating statistical significance.

## 3. Results

### 3.1. Model Predictive Performance and Assessment of Variable Importance

#### 3.1.1. Model Predictive Performance

Evaluation of the 11 single-algorithm models showed considerable variation in predictive performance among algorithms. RF achieved the highest mean AUC (0.95) and mean TSS (0.74), whereas SRE exhibited the weakest performance, with corresponding mean values of 0.65 and 0.30, respectively (Figure S1; Table S2). However, ensemble model construction was based on the performance of individual model runs rather than algorithm-level mean statistics. Specifically, only runs with TSS > 0.75 and AUC > 0.9 were retained for ensemble forecasting, whereas the values reported in Figure S1 represent averages across all runs. Consequently, inclusion in the ensemble model depended on whether individual runs satisfied the predefined evaluation thresholds, irrespective of the average performance of a given algorithm.

All four ensemble models (EMwmean, EMca, EMmean, and EMmedian) achieved substantially higher predictive accuracy, with AUC values exceeding 0.96 and TSS values above 0.83. Among them, EMwmean and EMca demonstrated the overall best performance. However, because EMwmean integrates model predictions using weighted probabilities, allowing for the quantification of the relative importance of contributing factors and providing more precise support for decision-making, it exhibited greater overall stability than the EMca approach. Therefore, the EMwmean ensemble model was selected for predicting current and future habitat suitability in this study.

#### 3.1.2. Importance of Environmental Variables

Because the ensemble model performed best, variable importance was evaluated using the results from 10 repeated model runs. The dominant environmental variables influencing the distribution of *Calliptamus italicus* were identified, in descending order of importance, as altitude, bio10 (mean temperature of the warmest quarter), bio18 (precipitation of the warmest quarter), bio9 (mean temperature of the driest quarter), bio17 (precipitation of the wettest quarter), and the global human footprint (GHF) (Table 1).

**Figure 3:**
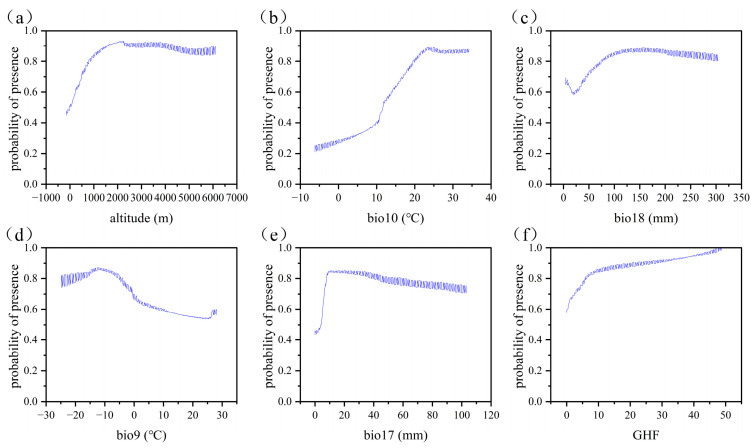
Influence of six key environmental variables on the survival probability of *Calliptamus italicus*: (**a**) altitude; (**b**) bio10 (mean temperature of the warmest quarter); (**c**) bio18 (precipitation of the warmest quarter); (**d**) bio9 (mean temperature of the driest quarter); (**e**) bio17 (precipitation of the driest quarter); and (**f**) GHF (Global Human Footprint).

To illustrate the probability of occurrence of *C. italicus* across different environmental gradients and to identify the optimal ranges of the key variables associated with habitat suitability, response curves were constructed to show how occurrence probability varies with major environmental variables (Figure 3).

When the probability of occurrence of *Calliptamus italicus* was ≥0.6, the response curves revealed clear relationships between habitat suitability and key environmental variables (Figure 3). As shown in Figure 3a, occurrence probability increased rapidly with elevation and reached a maximum value of approximately 0.9 between 1000 and 2000 m. Occurrence probability remained relatively high between 500 and 3000 m. The response curve for bio10 (mean temperature of the warmest quarter; Figure 3b) showed that occurrence probability increased with temperature and peaked between 20 °C and 25 °C, reaching values close to 0.9. When temperatures exceeded 30 °C, occurrence probability stabilized or decreased slightly. The precipitation response curve for the warmest season (bio18; Figure 3c) indicated optimal conditions within the 75–150 mm range, where species occurrence probability exceeded 0.8. At higher precipitation levels (>200 mm), probabilities tended to level off or slightly decrease. For precipitation of the driest quarter (bio17; Figure 3d), occurrence probability increased rapidly from 0 to 10 mm (from approximately 0.45 to 0.85) and declined slightly when precipitation exceeded 40 mm, with the range of 10–40 mm representing relatively high suitability (>0.8). As shown in Figure 3e, the response curve for the mean temperature of the coldest quarter (bio9) indicated the highest occurrence probabilities (0.8–0.9) within the range of −20 to −5 °C, followed by a decline as temperatures increased above 0 °C. The response curve for the global human footprint (GHF; Figure 3f) showed a positive relationship between human activity intensity and occurrence probability. The probability increased rapidly within the GHF range of 5–10 (from approximately 0.68 to 0.80) and continued to increase more gradually at values above 10 (0.83–1.00).

### 3.2. Prediction of Habitat Suitability for Calliptamus italicus

#### 3.2.1. Current Habitat Suitability in Xinjiang, China, and Kazakhstan

Based on predictions from the Biomod2 ensemble model, the total potential suitable habitat of *Calliptamus italicus*—including highly, moderately, and low suitability—covered approximately 4.28 × 10^5^ km^2^, accounting for 9.65% of the total study area (Figure S2). Among these, highly suitable areas covered 3.85 × 10^4^ km^2^ (0.87% of the study area), moderately suitable areas covered 16.67 × 10^4^ km^2^, and low suitable areas covered 22.28 × 10^4^ km^2^. In Xinjiang, China, high-suitability areas were mainly distributed along the Tianshan Mountains, particularly in Ili Prefecture, as well as the northwestern and southern parts of Tacheng Prefecture and the northern and northeastern parts of Altay Prefecture. Low suitability areas are mainly located in the northern part of Aksu Prefecture, the central part of Tacheng Prefecture, the southern part of Altay Prefecture, the central part of Changji Prefecture, and the western part of Hami City. In Kazakhstan, Low suitable habitats are mainly distributed in the southeastern Almaty Region, central Jetisu Region, southeastern Abai Region, central and northern East Kazakhstan Region, and the central Ulytau Region.

#### 3.2.2. Predictions of Habitat Suitability in Xinjiang, China, and Kazakhstan

**Figure 4:**
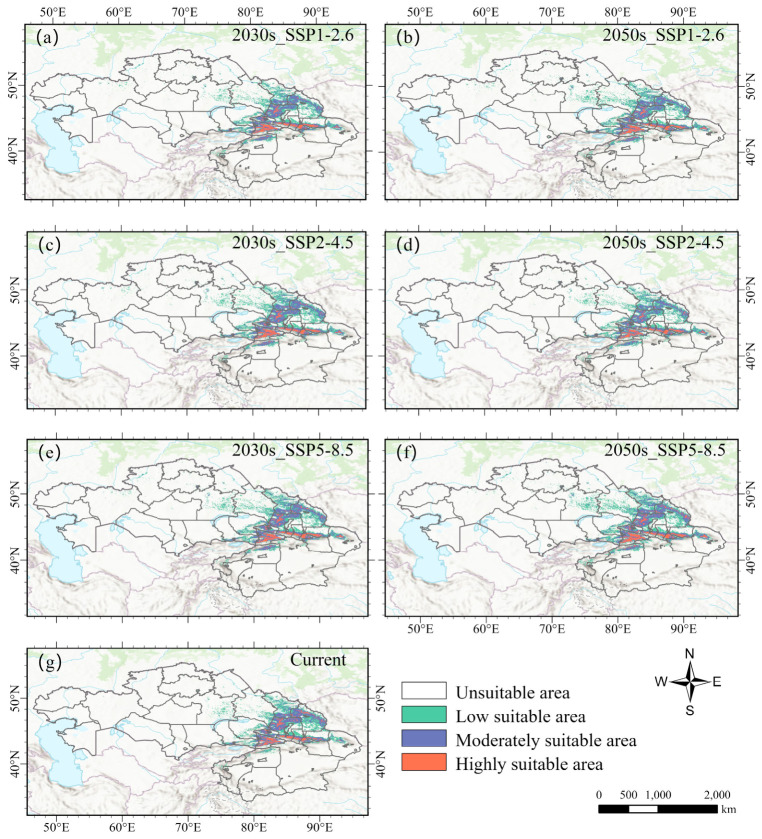
Potential suitable areas for *Calliptamus italicus* under low forcing scenario SSP1-2.6 in 2021–2040 (**a**) and 2041–2060 (**b**); Potential suitable areas for *Calliptamus italicus* under medium forcing scenario SSP2-4.5 in 2021–2040 (**c**) and 2041–2060 (**d**); Potential suitable areas for *Calliptamus italicus* under high forcing scenario SSP5-8.5 in 2021–2040 (**e**) and 2041–2060 (**f**); Potential suitable areas for *Calliptamus italicus* under current climatic conditions (**g**).

Under different Shared Socioeconomic Pathway (SSP) scenarios, future climate change is projected to induce moderate adjustments in the extent of suitable habitats for *Calliptamus italicus*, while the overall magnitude of change remains relatively small (Figure 4 and Figure S4, Table 2). In both the 2030s and 2050s, the area of unsuitable habitat exhibits an overall declining trend, with reductions ranging from approximately 0.49% to 2.52%. Correspondingly, the total area of suitable habitat—including highly, moderately, and low suitability—shows a slight expansion. Among these, low-suitability habitats display the largest relative increase, whereas changes in moderately and highly suitable areas are comparatively limited.

Under the SSP1-2.6 scenario (Figure 4a,b), the area of unsuitable habitat decreases to 398.63 × 10^4^ km^2^ (−0.49%) in the 2030s and further declines to 396.02 × 10^4^ km^2^ (−1.14%) in the 2050s. During the same periods, the area of low-suitability habitat increases to 24.13 × 10^4^ km^2^ (8.31%) and 26.25 × 10^4^ km^2^ (17.87%), respectively. In contrast, changes in medium- and high-suitability habitats are relatively limited, with area variation rates ranging from −0.13% to 2.48% and from 0.25% to 4.92%, respectively.

Under the SSP2-4.5 scenario (Figure 4c,d), unsuitable habitat decreases to 395.40 × 10^4^ km^2^ (−1.30%) in the 2030s, while the areas of low-, medium-, and high-suitability habitats increase to 26.11 × 10^4^ km^2^ (17.22%), 17.64 × 10^4^ km^2^ (5.15%), and 4.35 × 10^4^ km^2^ (12.99%), respectively. By the 2050s, however, unsuitable habitat slightly increases to 401.48 × 10^4^ km^2^ (0.22%), accompanied by reductions in medium- and high-suitability habitats to 15.13 × 10^4^ km^2^ (−9.81%) and 3.34 × 10^4^ km^2^ (−13.16%).

Under the SSP5-8.5 scenario (Figure 4e,f), unsuitable habitat decreases to 395.63 × 10^4^ km^2^ (−1.24%) in the 2030s, while low-, medium-, and high-suitability habitats expand to 25.75 × 10^4^ km^2^ (15.60%), 17.71 × 10^4^ km^2^ (5.55%), and 4.41 × 10^4^ km^2^ (14.59%), respectively. By the 2050s, unsuitable habitat further declines to 390.51 × 10^4^ km^2^ (−2.52%), and the areas of low-, medium-, and high-suitability habitats increase to 29.12 × 10^4^ km^2^ (30.73%), 18.67 × 10^4^ km^2^ (11.84%), and 5.11 × 10^4^ km^2^ (32.78%), respectively.

### 3.3. Quantification of Climatic Niche Dynamics of Calliptamus italicus Under Climate Change

To characterize climatic niche dynamics of *Calliptamus italicus* across present and future climate conditions, principal component analysis (PCA) was performed by integrating environmental variables associated with suitable habitats across all periods. The results showed that, across all climate scenarios and time periods, the explanatory power of the first two principal components (PC1 and PC2) remained highly consistent. Specifically, PC1 accounted for 22.3–22.5% of the total environmental variance, while PC2 explained 21.2–21.4%. Together, the first two principal components captured more than 43% of the overall environmental variation (Table S3), providing a stable environmental space for subsequent climatic niche dynamic analyses.

**Figure 5:**
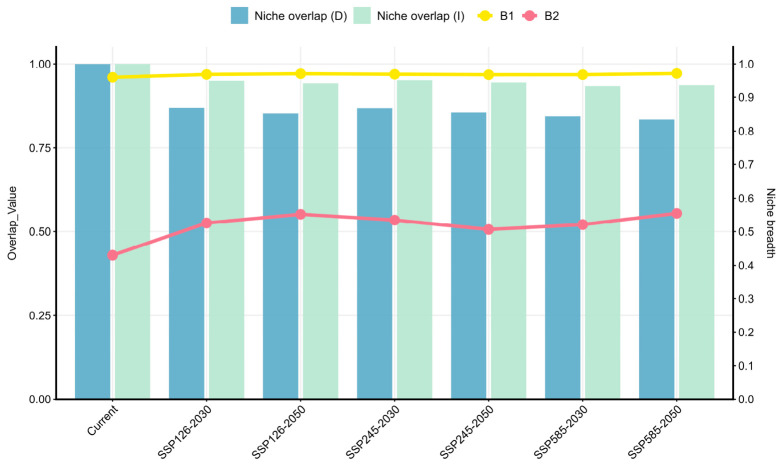
Ecological and geographic range overlap and ecological niche width of *Calliptamus italicus* in different periods and scenarios.

Levins’ niche breadth indices indicated that *C. italicus* consistently maintained a broad climatic niche across the entire study region (Table S3; Figure 5). Under all climate scenarios, Levins’ B1 values remained high (B1 > 0.95), indicating a relatively broad distribution of suitable habitats across the study region. Compared with the current climate, B1 values under future scenarios were slightly higher, indicating no substantial reduction in niche breadth under projected climate conditions. In contrast, the current Levins’ B2 value was relatively low (0.429), whereas B2 values under future climate scenarios increased to 0.506–0.554, reflecting a broader occupation of environmental space. The combined patterns of Levins’ B1 and B2 suggest an overall expansion of the climatic niche of *C. italicus* in both geographic and environmental dimensions under projected climate change, with the largest increase observed under high-emission scenarios by the 2050s.

**Figure 6:**
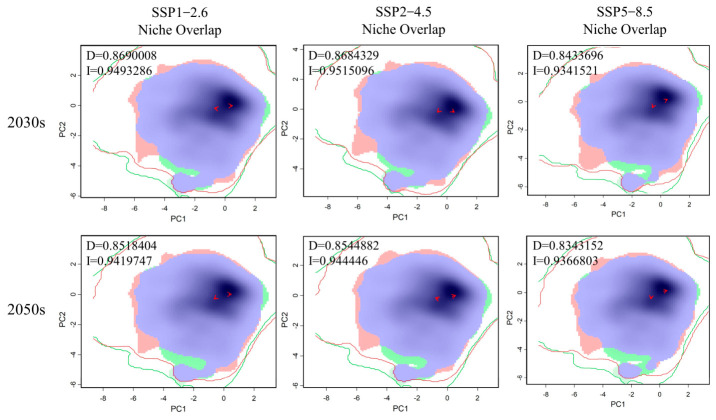
Ecological niche overlap and dynamics of climatic ecological niches of *Calliptamus italicus* (blue segments indicate stabilization, green segments decrease and red segments expansion). Note: The red and green lines represent 100% and 95% of the available environment, respectively. The purple area indicates the niche overlap between the two climate scenarios (niche stability), the red area represents the niche expansion under future climate scenarios (niche expansion), and the green area shows portions of the current niche that are unoccupied under future climate scenarios (niche loss). The solid red arrows and dashed red arrows denote the shifts of the species centroid and environmental centroid under the current and future climate scenarios, respectively.

Climatic niche overlap indices were evaluated to examine how *C. italicus* utilizes environmental resources across various periods and climate scenarios. Over time, Schoener’s D and the Hellinger I statistic exhibited a minor reduction in value within each scenario (Figure 5 and Figure 6; Table 3). Across future climate scenarios, Schoener’s D values ranged from 0.834 to 0.869, and I value ranged from 0.934 to 0.952, indicating a high degree of climatic niche overlap between projected future and present-day conditions.

Analysis of pairwise climatic niche comparisons for *C. italicus* demonstrated that the null hypothesis of niche equivalency was rejected in all cases (*p* < 0.05), except for the comparison between the current period and the 2050s under the SSP5-8.5 scenario (Table 3). In contrast, the null hypothesis of niche similarity was rejected for all comparisons (*p* < 0.05), indicating that the observed niche overlap patterns were significantly different from those expected under random niche allocation. The partitioning of climatic niche dynamics further showed that the future climatic niche of *C. italicus* is overwhelmingly dominated by niche stability, with stability proportions exceeding 99% across all scenarios. However, with increasing greenhouse gas emission levels and advancing time periods, niche expansion exhibited a slight increasing trend, accompanied by a marginal decline in niche stability.

## 4. Discussion

### 4.1. Key Environmental Variables Influencing the Distribution of Calliptamus italicus

*Calliptamus italicus* is a characteristic grassland locust widely distributed in the temperate drylands of Eurasia, particularly in semi-arid steppes, desert-steppes, and agricultural–pastoral ecotones. Previous studies have shown that the species prefers open habitats with sparse vegetation, loose soils suitable for oviposition, and relatively warm and dry climatic conditions. The present model identified altitude, temperature, precipitation, and human footprint as the primary environmental determinants of its distribution. Overall, these predictors are highly consistent with the known ecological requirements of the species and help explain its concentration in the dry grassland ecosystems of Xinjiang, China and Central Asia [22].

Regarding topographic factors, *C. italicus* exhibited the highest occurrence probability within the altitudinal range of approximately 1000–2000 m and maintained relatively high habitat suitability between 500 and 3000 m. These results align with field observations showing that major outbreak areas of *C. italicus* in Central Asia and Xinjiang, China are primarily located on low- to mid-elevation hills, piedmont plains, and the margins of plateaus [7,49]. Mid-elevation regions are often associated with relatively moderate climatic conditions and extensive grassland habitats, which may contribute to the high occurrence probabilities observed in these areas [50]. Notably, even at elevations exceeding 2000 m, *C. italicus* maintained a relatively high probability of occurrence, suggesting a certain degree of tolerance to low-temperature conditions [51].

Temperature was identified as another key determinant of distribution. Summer mean temperature (bio10) within the range of approximately 20–25 °C was most favorable for *C. italicus*, aligning well with the optimal thermal conditions for population growth in Orthopteran insects, which are generally characterized by high survival rates, enhanced reproductive performance, and shortened generation times around 24 °C [52]. When summer temperatures exceeded this range, the occurrence probability tended to stabilize or slightly decline, potentially due to intensified evapotranspiration, increased vegetation water stress, and heightened risks of thermal stress to nymphs under extreme heat conditions [53].

Precipitation-related variables further corroborated the species’ adaptation to arid environments. Higher occurrence probabilities were observed under relatively low summer precipitation and low precipitation during the driest quarter, whereas habitat suitability slightly declined under high precipitation conditions. These results indicate that *C. italicus* exhibits strong tolerance to low-precipitation and high-evaporation environments and preferentially occupies semi-arid to arid grassland habitats. This pattern corresponds well with the distribution of its primary host plants, such as drought-tolerant Artemisia species and xerophytic grasses [54]. Excessive precipitation may lead to overly dense vegetation and elevated soil moisture, thereby reducing habitat suitability for oviposition and early developmental stages.

Global human footprint (GHF) showed a significant positive relationship with the occurrence probability of *C. italicus*. However, GHF reflects multiple dimensions of human influence on landscapes and ecosystems, making it difficult to identify the specific mechanisms underlying its association with species occurrence. Consequently, the positive correlation identified by our SDM does not imply a causal relationship and should not be interpreted as evidence that human activities directly promote the occurrence of *C. italicus*. Instead, the observed association may reflect indirect environmental changes associated with human land use, shared responses of both GHF and species occurrence to other environmental variables, or historical overlap between areas of intensive human activity and habitats naturally suitable for the species. Thus, while the observed pattern aligns with long-term observations reported by FAO Locust Watch, causation cannot be inferred from the correlative modelling framework used in this study [7,55]. Nonetheless, areas characterized by relatively high GHF values and suitable climatic conditions were consistently associated with higher predicted occurrence probabilities and may therefore warrant additional attention in future monitoring and risk management programs.

### 4.2. Future Distribution Shifts of Calliptamus italicus Under Climate Change

Future climate projections indicate that the suitable habitat of *Calliptamus italicus* will gradually expand from 2030 to 2050, although the extent and rate of expansion vary among different Shared Socioeconomic Pathways (SSPs). The SSP1-2.6 scenario predicts a smaller expansion of the range. This may be because relatively moderate warming is unlikely to substantially alter moisture conditions in arid and semi-arid regions, while vegetation phenological changes remain relatively mild [56,57]. Consequently, plant biomass in newly suitable areas under the low-emission scenario may be insufficient to support successful population establishment [58,59]. Under the SSP2-4.5 scenario, the suitable habitat expands by 2030 but contracts by 2050. This pattern may occur because climatic conditions become increasingly favorable for the growth and development of *C. italicus* by 2030, thereby enhancing population persistence and overwintering success [60]. However, continued warming by 2050 may expose some regions to greater thermal stress during the warm season. Experimental studies have shown that survival rate and population growth parameters may decline when individuals are exposed to temperatures around 35 °C [50], suggesting that prolonged exposure to high temperatures can negatively affect physiological performance [61]. It is important to note, however, that these experimental observations represent short-term physiological responses measured under controlled temperature conditions, whereas the SDM predicts long-term climatic suitability based on broad-scale environmental averages. Accordingly, the relatively high occurrence probabilities associated with elevated bio10 values (Figure 3b) do not necessarily imply that individuals experience no thermal stress. Rather, they indicate that regions with warmer growing seasons may remain suitable overall when multiple climatic factors are considered simultaneously. Because bio10 represents the mean temperature of the warmest quarter rather than short-term temperature extremes, occasional or seasonal heat stress may still occur within climatically suitable areas. Therefore, the physiological limitations observed in laboratory studies and the habitat suitability patterns predicted by the SDM reflect different ecological processes operating at different temporal scales and are not inherently contradictory. Under the SSP5-8.5 scenario, the model predicts a much greater habitat expansion, likely due to more pronounced climate warming, which could transform larger areas previously unsuitable due to low temperatures—such as high-altitude mountainous regions and high-latitude grasslands—into potential habitats for *C. italicus* [62]. Furthermore, SSP5-8.5 is projected to experience the greatest changes in temperature and precipitation regimes. These climatic changes may contribute to the larger expansion predicted by the model [63]. Vast climatically suitable areas may become increasingly connected under future warming, potentially enhancing habitat continuity and facilitating regional dispersal of *C. italicus* across adjacent grassland ecosystems [64,65].

However, continued warming by 2050 may expose some regions to greater thermal stress during the warm season. Experimental studies have shown that survival rate and population growth parameters may decline when individuals are exposed to temperatures around 35 °C [50], suggesting that prolonged heat stress could negatively affect physiological performance and population maintenance. It should be noted, however, that the response curve of bio10 remained relatively high at elevated temperatures (Figure 3b). This apparent discrepancy likely arises because bio10 represents the mean temperature of the warmest quarter rather than short-term temperature extremes. Therefore, physiological responses observed under constant high-temperature conditions do not necessarily contradict the high occurrence probabilities predicted by the SDM, which reflect long-term habitat suitability.

Under the SSP5-8.5 scenario, the model predicts a substantially greater expansion of suitable habitat, likely driven by more pronounced climate warming. Rising temperatures may render previously unsuitable areas, such as high-altitude mountainous regions and high-latitude grasslands, increasingly favorable for the establishment of *C. italicus* [51]. Furthermore, the frequency and intensity of extreme climatic events are projected to increase under this scenario [52]. Although such events may impose local physiological stress, the overall expansion of climatically suitable conditions is predicted to outweigh these negative effects at the spatial scale considered by the model. As a result, vast suitable areas may become increasingly connected under future warming, potentially enhancing habitat continuity and facilitating regional dispersal of *C. italicus* across adjacent grassland ecosystems [53,54].

**Figure 7:**
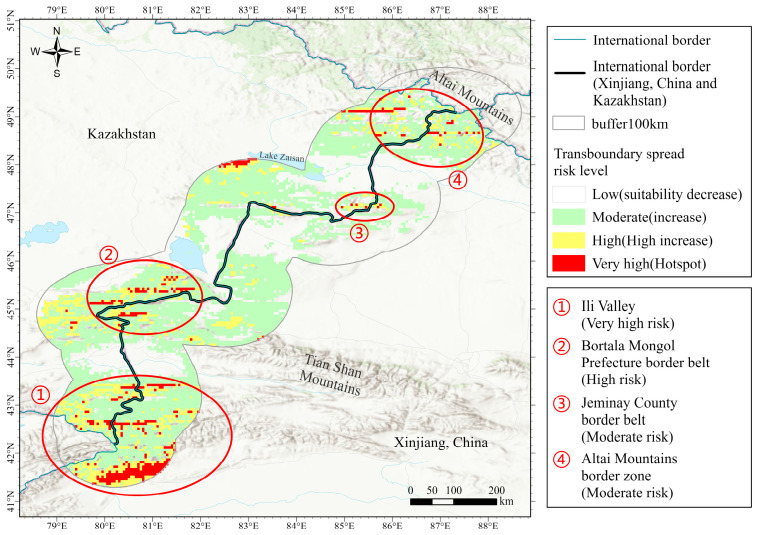
Spatial hotspots of transboundary spread risk of *Calliptamus italicus* under SSP5-8.5 in the 2050s.

Within the 100-km buffer zone along the border, we found several areas prone to pest infestations, with a particularly high concentration in the Ili River Valley (Figure 7). The Ili River Valley has long served as a vital geographical corridor connecting Xinjiang, China and Central Asia. The contiguous distribution of grasslands and river valleys within the region may weaken geographical barriers, facilitating the migration and spread of *C. italicus*. Against the backdrop of future climate warming, the increasing climatic suitability of border regions may further heighten the risk of this species’ regional spread and cross-border expansion [64,66].

The increase in suitable habitat within the border buffer zone suggests that future climate change may reduce climatic barriers between Xinjiang, China and Kazakhstan, thereby creating more favorable conditions for the regional spread and potential transboundary expansion of *C. italicus* (Table S4). However, the response of *C. italicus* to future climate change is not entirely linear. The reduction in suitable habitat under the SSP2-4.5 scenario in the 2050s suggests that moderate warming does not always favor habitat expansion in arid ecosystems. Excessive heat stress, declining soil moisture or intensified drought may offset the positive effects of warming in certain regions. This finding highlights the complex interactions between temperature and water availability in shaping the future distribution patterns of xerophilic insects [67].

However, our findings differ somewhat from those of some recent studies. For example, Wu et al. (2022) [20], using a MaxEnt-based approach, predicted that the overall suitable habitat of three typical locust species (*C. italicus*, *Locusta migratoria*, and *Gomphocerus sibiricus*) in Xinjiang, China and Kazakhstan would decline in the future, although highly suitable habitats would temporarily expand during the near term (2030s) before decreasing, thereby increasing localized outbreak risks. Such discrepancies may arise from several factors. Regarding spatial resolution, Wu used a spatial resolution of 0.5°, which is suitable for identifying broad-scale continental trends but may obscure critical topographic barriers and valley-scale microclimatic features in mountainous regions of Central Asia, such as the Tianshan and Altay ranges [68,69]. Regarding the selection of environmental variables, in addition to climatic factors, our study incorporated high-resolution soil data and human footprint data. This approach is better suited for identifying small-scale suitable habitats and more closely reflects the ecological reality of *C. italicus*, particularly its soil-dependent oviposition behavior and strong association with agricultural activities [70,71]. In terms of research focus, Wu simultaneously modeled multiple representative locust species, emphasizing shared climatic constraints at the regional scale while potentially underrepresenting species-specific niche requirements [72,73,74]. Earlier research indicates that *C. italicus* is well adapted to arid and semi-arid environments, with distributions closely associated with desert steppes, typical grasslands, and agro-pastoral transition zones, and shows relatively broad tolerance to increasing temperatures and decreasing precipitation [3,50,75]. Under such conditions, when future climate change is characterized primarily by warming and altered precipitation regimes, the potential distribution of *C. italicus* may not necessarily contract uniformly and may instead expand in certain climatically marginal areas [76].

### 4.3. Climatic Niche Conservatism of Calliptamus italicus Under Future Climate Change

The principal component analysis (PCA) showed that, across different climate scenarios and time periods, the first two principal components (PC1 and PC2) consistently explained a stable proportion of the total environmental variance. This indicates that although future climate conditions are projected to change, the key climatic gradients governing the distribution of *Calliptamus italicus* in the study area have not undergone fundamental restructuring, but rather changes in intensity. Such stability in PCA structure is regarded as a prerequisite for conducting cross-temporal and cross-scenario niche comparisons in climatic niche dynamics studies [48,77]. Ecologically, PC1 and PC2 jointly represent temperature and moisture gradients, and the minimal variation in their explanatory power among scenarios suggests that the climatic constraints faced by *C. italicus* remain primarily dominated by the classic “heat–water” gradient. This pattern is highly consistent with the species’ ecological characteristics as a locust adapted to arid and semi-arid grassland environments [78].

Niche overlap analyses further demonstrated that Schoener’s D and I values remain relatively high under future climate scenarios, indicating a strong overlap between future and current climatic niches and thus pronounced niche conservatism in *C. italicus* [79,80]. Although niche overlap showed a slight declining trend over time within the same scenario, the overall magnitude of change was limited. This suggests that climate warming may not substantially alter the ecological niche of *C. italicus*, but instead increase the geographic availability of habitats that match its existing climatic preferences [48]. In arid and semi-arid regions, grassland ecosystems often exhibit high climatic continuity across political boundaries. As climatically analogous habitats expand under warming conditions, climatic discontinuities between Xinjiang, China and eastern Kazakhstan may gradually decrease, facilitating stepwise range expansion across connected dryland landscapes [81]. In this context, niche conservatism may not restrict distributional change; instead, it may facilitate regional spread when future climates increasingly resemble the species’ existing niche conditions [79,80,82].

The increase in Levins’ B2 values under future climate scenarios further supports this interpretation. Compared with the present climate, future populations of *C. italicus* are projected to occupy a broader environmental space, suggesting increased tolerance to climatic heterogeneity. Greater environmental tolerance may improve the ability of populations to establish and persist in marginal border regions, thereby enhancing the stability of newly colonized habitats [83,84].

Therefore, the future spread of *C. italicus* may primarily depend on increasing habitat connectivity and climatic continuity rather than rapid ecological adaptation. This finding highlights the importance of considering climatic niche conservatism when assessing future transboundary ecological risks in dryland pest species.

From a management perspective, this marked niche conservatism implies that the future risk of *C. italicus* proliferations will be concentrated primarily in regions where climatic conditions resemble those of their existing habitats. Consequently, as the climate warms, the risk of *C. italicus* expansion in farmland and natural grasslands within the climatic zones similar to those along the Xinjiang, China—Kazakhstan border will continue to rise. Specifically, conducting long-term monitoring in grassland ecosystems with suitable climatic conditions and strengthening cross-border cooperation on pest control will help mitigate the potential harm caused by locust infestations to crop production and the ecological stability of grasslands.

### 4.4. Implications for Locust Management Strategies

The results indicate that *Calliptamus italicus* maintains a relatively stable core climatic niche, and its potential suitable range is set to increase under future climate scenarios. Based on these spatially explicit projections, the following targeted management strategies are recommended.

First, a fine-scale and spatially prioritized monitoring network should be established. Monitoring efforts should focus on the projected centroid migration corridor, particularly from the Tianshan Mountains toward the Kazakhstan border, where newly suitable habitats are expected to emerge. Digital monitoring stations and remote sensing-based surveillance systems can be deployed to improve early detection capacity, especially in semi-natural habitats affected by human disturbance.

Second, ecological habitat management strategies should be implemented to reduce habitat suitability. As the species shows a clear preference for arid conditions and sparsely vegetated environments, measures such as grazing exclusion, vegetation restoration, and ground cover enhancement should be prioritized in high-risk areas to modify microhabitat conditions and disrupt egg overwintering success.

Third, the timing of biological control interventions should be optimized based on climatic thresholds. Given that the response curves indicate an optimal growth temperature of approximately 20–25 °C, environmentally friendly biocontrol agents (e.g., microsporidia) should be applied during this critical thermal window to maximize control efficiency and reduce unnecessary intervention costs.

Finally, transboundary collaborative management mechanisms should be strengthened. Establishing a long-term China–Kazakhstan joint monitoring framework, including shared databases, coordinated field surveys, and early-warning systems, is essential. Key regions, such as the Abai Region in Kazakhstan and northern Xinjiang in China, should be prioritized for joint prevention efforts to enhance regional biosecurity and build an integrated ecological defense system against locust outbreaks.

### 4.5. Limitations

Despite the robust performance of the ensemble modeling framework, several limitations should be acknowledged.

First, in areas with insufficient sampling, uneven geographical coverage within the study area may still introduce uncertainty into the predicted suitability distribution patterns; such biases are a common challenge in species distribution modelling [85,86]. Although our occurrence records are compiled from multiple sources and spatial filtering has been applied to reduce sampling bias, this may still introduce uncertainty regarding the characteristics of the relationship between species and their environment. To further evaluate the potential influence of geographic sampling bias, we additionally compiled occurrence records from the FAO CCALM (Caucasus and Central Asia Locust Management System, online GIS database: https://ccalm.org/engine, accessed on 11 June 2026) database and conducted a supplementary sensitivity analysis using an expanded dataset (1318 records after spatial filtering) (Figure S5). The resulting habitat suitability predictions were highly consistent with those generated from the original dataset (Figures S2 and S6). Within the 100-km transboundary buffer zone between Xinjiang, China and Kazakhstan, approximately 87% of the suitable habitat predicted by the expanded dataset was also identified by the original model, while the total suitable habitat area differed by only 11% (Table S6). These results indicate that the main conclusions of this study remain robust even when additional distribution records are not included.

Second, the models incorporated climatic, topographic, soil, and human-footprint variables, but some ecologically important factors were not explicitly considered. Previous studies have suggested that *C. italicus* is associated with steppe vegetation communities dominated by *Artemisia* spp., which provide suitable feeding and breeding habitats [7]. However, due to the limited availability of spatially explicit vegetation datasets and future vegetation projections, vegetation-related variables were not included in the present study. In addition, biotic interactions, such as natural enemies, parasitoids, competition, and density-dependent population regulation, were not considered [87]. The omission of these factors may contribute to localized prediction uncertainties and may partially explain differences between potential and realized distributions [88]. In future research, we will need to incorporate vegetation data (particularly information on the distribution of Artemisia communities and other host plants) in order to refine the habitat suitability assessment model.

Third, although multicollinearity analysis was performed prior to model construction, 19 environmental variables were ultimately retained. While ensemble modeling can reduce uncertainty associated with individual algorithms, the inclusion of a relatively large number of predictors may increase the risk of model overfitting and reduce predictive generality under novel environmental conditions [89]. Future studies could further evaluate model complexity and explore more parsimonious variable-selection approaches.

Finally, uncertainties associated with future climate projections remain unavoidable. Only a single CMIP6 general circulation model (BCC-CSM2-MR) was used to project future climatic suitability. Although this model performs reasonably well in arid and semi-arid regions of Central Asia, different climate models may produce varying estimates of future environmental conditions [7,90]. Furthermore, model evaluation was primarily based on internal cross-validation metrics, whereas independent validation datasets were unavailable [91]. Future studies should incorporate multi-model climate ensembles, vegetation dynamics, and independent occurrence datasets to improve the robustness and ecological realism of habitat suitability forecasts.

## 5. Conclusions

This study utilized the Biomod2 integrated species distribution model, combined with the Ecospat niche analysis method, to investigate the current status and future distribution dynamics of *Calliptamus italicus* in Xinjiang, China and Kazakhstan in the context of climate change. Temperature, precipitation, topography and human activities are the primary environmental drivers determining the distribution pattern of this species. Simulation results under different future climate scenarios indicate that the suitable habitat of *Calliptamus italicus* is expanding, with the greatest extent of habitat expansion observed under the high-emission scenario. The increase in high-risk pest areas in border regions and the continued improvement in habitat suitability indicate that climate warming will exacerbate the risk of cross-border spread of this species between Xinjiang, China, and Kazakhstan. Combined with climate niche analysis, the future expansion of the range of *Calliptamus italicus* is not driven by fundamental changes in its ecological niche, but rather by a gradual migration towards habitats with similar climatic conditions in a warming environment.

## Figures and Tables

**Table 1:** Information on the data sources.

Variable	Description	Variable Importance (%)	Sorting
altitude	Altitude	18.4	1
bio10	Mean Temperature of Warmest Quarter	13.9	2
bio18	Precipitation of Warmest Quarter	8.9	3
bio9	Mean Temperature of Driest Quarter	8.3	4
bio17	Precipitation of Driest Quarter	8.1	5
GHF	Global Human Footprint Data	7.7	6
aspect	Aspect	6.4	7
bio4	Temperature Seasonality	3.2	8
t_clay	Clay content of the topsoil layer (0~5 cm)	1.4	9
bio2	Mean Diurnal Range	1.3	10

**Table 2:** Future suitable area changes of *Calliptamus italicus*.

Climate Scenario	Period	Area of Unsuitable /(×10^4^ km^2^)	Change (%)	Area of Low Suitability /(×10^4^ km^2^)	Change (%)	Area of Moderate Suitability /(×10^4^ km^2^)	Change (%)	Area of High Suitability /(×10^4^ km^2^)	Change (%)
SSP1-2.6	Current	400.6		22.28		16.67		3.85	
	2030S	398.63	↓ 0.49	24.13	↑ 8.31	16.65	↓ 0.13	4.09	↑ 0.25
	2050S	396.02	↓ 1.14	26.25	↑ 17.87	17.19	↑ 2.48	4.03	↑ 4.92
SSP2-4.5	2030S	395.4	↓ 1.3	26.11	↑ 17.22	17.64	↑ 5.15	4.35	↑ 12.99
	2050S	401.48	↑ 0.22	23.55	↑ 5.71	15.13	↓ 9.81	3.34	↓ 13.16
SSP5-8.5	2030S	395.63	↓ 1.24	25.75	↑ 15.6	17.71	↑ 5.55	4.41	↑ 14.59
	2050S	390.51	↓ 2.52	29.12	↑ 30.73	18.67	↑ 11.84	5.11	↑ 32.78

Note: ↑ indicates an increase compared with the current period; ↓ indicates a decrease.

**Table 3:** Comparisons of ecological niches within the of potential distribution of *Calliptamus italicus* between the current and future climate scenarios.

	D	I	Equivalency (*P*)	Similarity (*P*)	Expansion (%)	Stability (%)	Unfilling (%)
Current vs. SSP1-2.6-2030S	0.8690008	0.9493286	0.00416	0.00083	0.67	99.33	0.27
Current vs. SSP1-2.6-2050S	0.8518404	0.9419747	0.01166	0.00167	0.68	99.31	0.43
Current vs. SSP2-4.5-2030S	0.8684329	0.9515096	0.00167	0.00083	0.74	99.26	0.24
Current vs. SSP2-4.5-2050S	0.8544882	0.944446	0.01415	0.00083	0.76	99.24	0.44
Current vs. SSP5-8.5-2030S	0.8433696	0.9341521	0.00583	0.00333	0.86	99.14	0.37
Current vs. SSP5-8.5-2050S	0.8343152	0.9366803	0.06828	0.00083	0.77	99.23	0.46

## Data Availability

The original contributions presented in this study are included in the article and Supplementary Material. Further inquiries can be directed to the corresponding author.

## Supplementary Materials

The following supporting information can be downloaded at: https://www.mdpi.com/article/10.3390/insects17070710/s1, Figure S1: AUC and TSS values for single models and ensemble models; Figure S2: Potential suitable areas for *Calliptamus italicus* under present climate scenarios; Figure S3: Area of suitable habitats under different carbon emission scenarios; Figure S4: Distribution of pseudo-presence points and existent points; Figure S5: Distribution points of *Calliptamus italicus* including those provided by FAO CCALM; Figure S6: Potential suitable areas for *Calliptamus italicus* under current climate conditions, predicted using occurrence data including those provided by FAO CCALM; Table S1: 19 environmental factors affecting the distribution of *Calliptamus italicus*; Table S2: Mean and standard deviation of AUC and TSS values for the 11 individual algorithms across repeated model runs; Table S3: Ecological niche width and variation in principle components PC1 and PC2 between current and future projected distribution range; Table S4: Changes in suitable habitat area within the China–Kazakhstan border buffer under future climate scenarios; Table S5: Potential environmental factor affecting the distribution of *Calliptamus italicus*; Table S6: Comparison of predicted suitable habitats within the 100 km Xinjiang, China–Kazakhstan border buffer based on the original and FAO CCALM-expanded occurrence datasets.

## References

[1] Lachininsky A.V., Sergeev M.G., Childebaev M.K., Chernyakhovsky M.E., Lockwood J.A., Kambulin V.E., Gapparov F.A. (2002). Locusts of Kazakhstan, Central Asia and Adjacent Territories.

[2] Sergeev M.G. (1992). Distribution patterns of orthoptera in North and Central Asia. J. Orthoptera Res..

[3] Sergeev M., Childebaev M., Vankova I., Gapparov F., Kambulin V., Kokanova E., Lachininsky A., Pshenitsyna L., Temreshev I., Chernyakhovsky M. (2022). Italian Locust Calliptamus italicus (Linnaeus, 1758).

[4] Lecoq M., Zhang L. (2019). Encyclopedia of Pest Orthoptera of the World.

[5] Sergeev M.G. (2021). Ups and downs of the Italian locust (*Calliptamus italicus* L.) populations in the Siberian steppes: On the horns of dilemmas. Agronomy.

[6] Azhbenov V. (2000). Outbreaks and migrations of locusts in Kazakstan. Stepnoj Bull..

[7] Sergeev M.G., Childebaev M.K., Ji R., Molodtsov V.V., Baturina N.S., Van’kova I.A., Kim-Kashmenskaya M.N., Popova K.V., Zharkov V.D., Yefremova O.V. (2025). Ecologo-Geographic Distribution Patterns of the Italian Locust *Calliptamus italicus* (Linnaeus) (Orthoptera: Acrididae) in the Easternmost Part of Its Range. Insects.

[8] Vasil’ev K. (1962). Italian locust (*Calliptamus italicus*) in Central Kazakhstan. Proc. All-Union Inst. Plant Prot..

[9] Sergeev M.G., Van’kova I. (2008). The dynamics of a local population of the Italian locust (*Calliptatus italicus* L.) in an anthropogenic landscape. Contemp. Probl. Ecol..

[10] Li H.-C., Xia K.-L., Bi D., Jin X., Huang C., Yin X., Zheng Z., Liang Z., You Q., Zhang F. (2006). Fauna Sinica, Insecta, Vol. 43, Orthoptera, Acridoidea, Catantopidae. Sci. Press Seijing.

[11] Guo H., Wu L., Zhao L. (2011). A brief discussion on locust damage and control strategies in Tacheng border region. Xinjiang Anim. Husb..

[12] Liu Q., He L.Z., Zhang Y.J. (2017). Important ecological characteristics of locust breeding and outbreak areas along the China–Kazakhstan border. J. Environ. Entomol..

[13] Lee H., Calvin K., Dasgupta D., Krinner G., Mukherji A., Thorne P., Trisos C., Romero J., Aldunce P., Barret K., Lee H., Romero J., Core Writing Team (2023). IPCC, 2023: Climate Change 2023: Synthesis Report, Summary for Policymakers. Contribution of Working Groups I, II and III to the Sixth Assessment Report of the Intergovernmental Panel on Climate Change.

[14] Grünig M., Mazzi D., Calanca P., Karger D.N., Pellissier L. (2020). Crop and forest pest metawebs shift towards increased linkage and suitability overlap under climate change. Commun. Biol..

[15] Darvishzadeh A., Bandani A.R. (2012). Identification and characterization of alpha-amylase in the Italian locust, *Calliptamus italicus* (Linnaeus, 1758) (Orthoptera: Acrididae). Mun. Ent. Zool..

[16] Malekmohammadi A., Shishehbor P., Kocheili F. (2012). Influence of constant temperatures on development, reproduction and life table parameters of *Encarsia inaron* (Hymenoptera: Aphelinidae) parasitizing *Neomaskellia andropogonis* (Hemiptera: Aleyrodidae). Crop Prot..

[17] Pacifici M., Visconti P., Butchart S.H., Watson J.E., Cassola F.M., Rondinini C. (2017). Species’ traits influenced their response to recent climate change. Nat. Clim. Change.

[18] Thuiller W., Lafourcade B., Engler R., Araújo M.B. (2009). BIOMOD–a platform for ensemble forecasting of species distributions. Ecography.

[19] Marmion M., Parviainen M., Luoto M., Heikkinen R.K., Thuiller W. (2009). Evaluation of consensus methods in predictive species distribution modelling. Divers. Distrib..

[20] Wu R., Guan J.-Y., Wu J.-G., Ju X.-F., An Q.-H., Zheng J.-H. (2022). Predictions based on different climate change scenarios: The habitat of typical locust species is shrinking in Kazakhstan and Xinjiang, China. Insects.

[21] Guo X., Lin J., Zheng J., Zhang F., Li X., Liu L., Liu X., Wu J., Wang N., Zhang K. (2026). Risk assessment of transboundary locust habitat distribution and migration pathways under climate change: A case study of Kazakhstan and Xinjiang, China. Geomat. Nat. Hazards Risk.

[22] Klein I., van der Woude S., Schwarzenbacher F., Muratova N., Slagter B., Malakhov D., Oppelt N., Kuenzer C. (2022). Predicting suitable breeding areas for different locust species–A multi-scale approach accounting for environmental conditions and current land cover situation. Int. J. Appl. Earth Obs. Geoinf..

[23] Zhang F., Guo X., Zheng J., Lin J., Li X., Liu L., Han W., Li W., Yan Y., Luo J. (2026). Comparative modelling of two migratory locusts along the China–Kazakhstan border under climate change: Poleward habitat shifts and increasing transboundary risk. Pest Manag. Sci..

[24] GBIF.org (2025). GBIF Occurrence Download. https://doi.org/10.15468/dL.4rwzhz.

[25] Dandabathula G., Hari R., Ghosh K., Fararoda R., Kumare D., Sasikumar A., Bera A.K., Srivastav S.K. (2023). Geospatial Analysis for Determination of Preferential Soil Conditions for The Desert Locust Oviposition. Eur. J. Ecol..

[26] Wu T., Hao S., Kang L. (2021). Effects of soil temperature and moisture on the development and survival of grasshopper eggs in inner mongolian grasslands. Front. Ecol. Evol..

[27] Mukerji M., Gage S. (1978). A model for estimating hatch and mortality of grasshopper egg populations based on soil moisture and heat. Ann. Entomol. Soc. Am..

[28] Edwards R.L., Epp H.T. (1965). The influence of soil moisture and soil type on the oviposition behaviour of the migratory grasshopper, *Melanoplus sanguinipes* (Fabricius). Can. Entomol..

[29] Hijmans R.J., Phillips S., Leathwick J., Elith J., Hijmans M.R.J. (2017). Package ‘dismo’. Circles.

[30] Mu H., Li X., Wen Y., Huang J., Du P., Su W., Miao S., Geng M. (2022). A global record of annual terrestrial Human Footprint dataset from 2000 to 2018. Sci. Data.

[31] Li S., Quan W., Wang Z., Chen Y., Yan P. (2023). Evaluation of the ability of BCC-CSM2-MR global climate model in simulating precipitation and temperature in East Asia. J. Arid. Meteorol..

[32] Wu T., Lu Y., Fang Y., Xin X., Li L., Li W., Jie W., Zhang J., Liu Y., Zhang L. (2019). The Beijing climate center climate system model (BCC-CSM): The main progress from CMIP5 to CMIP6. Geosci. Model Dev..

[33] Pei Y., Song M., Ma X., Wu T., Zhang S. (2022). Simulation assessment and prediction of future temperatures in Northwest China from BCC-CSM Model. Sci. Cold Arid. Reg..

[34] Low B.W., Zeng Y., Tan H.H., Yeo D.C. (2021). Predictor complexity and feature selection affect Maxent model transferability: Evidence from global freshwater invasive species. Divers. Distrib..

[35] Pearce J., Ferrier S. (2000). An evaluation of alternative algorithms for fitting species distribution models using logistic regression. Ecol. Model..

[36] Mohamed Nisin K.M.N., Sreenath K., Sreeram M.P. (2024). Muscling mussels: Understanding the invasive potential of the South American bivalve *Mytella strigata* (Hanley, 1843) in the Northern Indian Ocean. Sci. Total Environ..

[37] Wang D., Shi C., Alamgir K., Kwon S., Pan L., Zhu Y., Yang X. (2022). Global assessment of the distribution and conservation status of a key medicinal plant (*Artemisia annua* L.): The roles of climate and anthropogenic activities. Sci. Total Environ..

[38] Wang Y.S., Xie B.Y., Wan F.H. (2007). Application of ROC curve analysis in evaluating invasive species distribution models. Biodivers. Sci..

[39] Swets J.A. (1988). Measuring the accuracy of diagnostic systems. Science.

[40] Allouche O., Tsoar A., Kadmon R. (2006). Assessing the accuracy of species distribution models: Prevalence, kappa and the true skill statistic (TSS). J. Appl. Ecol..

[41] Jiménez-Valverde A. (2011). Opinion: Relationship between local population density and environmental suitability estimated from occurrence data. Front. Biogeogr..

[42] Liu C., Berry P.M., Dawson T.P., Pearson R.G. (2005). Selecting thresholds of occurrence in the prediction of species distributions. Ecography.

[43] Warren D.L., Glor R.E., Turelli M. (2008). Environmental niche equivalency versus conservatism: Quantitative approaches to niche evolution. Evolution.

[44] Warren D.L., Glor R.E., Turelli M. (2010). ENMTools: A toolbox for comparative studies of environmental niche models. Ecography.

[45] Cai Q., Welk E., Ji C., Fang W., Sabatini F.M., Zhu J., Zhu J., Tang Z., Attorre F., Campos J.A. (2021). The relationship between niche breadth and range size of beech (Fagus) species worldwide. J. Biogeogr..

[46] Slatyer R.A., Hirst M., Sexton J.P. (2013). Niche breadth predicts geographical range size: A general ecological pattern. Ecol. Lett..

[47] Warren D.L., Seifert S.N. (2011). Ecological niche modeling in Maxent: The importance of model complexity and the performance of model selection criteria. Ecol. Appl..

[48] Broennimann O., Fitzpatrick M.C., Pearman P.B., Petitpierre B., Pellissier L., Yoccoz N.G., Thuiller W., Fortin M.J., Randin C., Zimmermann N.E. (2012). Measuring ecological niche overlap from occurrence and spatial environmental data. Glob. Ecol. Biogeogr..

[49] Luo D., Liu Q., Wang J., Jashenko R., Ji R. (2023). Transcriptome analysis of the differentially expressed heat-resistant genes between *Calliptamus italicus* and Gomphocerus sibiricus. Environ. Entomol..

[50] Uvarov B. (1977). Grasshoppers and Locusts. A Handbook of General Acridology. Behaviour, Ecology, Biogeography, Population Dynamics.

[51] Li Y., Liu Q., Zhang X., Mao B., Yang G., Shi F., Bi J., Ma Z., Tang G. (2024). Effects of environmental factors on the diversity of grasshopper communities along altitude gradients in Xizang, China. Insects.

[52] Li W.-b., Gao Y., Cui J., Shi S.-S. (2020). Effects of temperature on the development and fecundity of *Atractomorpha sinensis* (Orthoptera: Pyrgomorphidae). J. Econ. Entomol..

[53] Angilletta M.J. (2010). Thermal adaptation: A theoretical and empirical analysis. Integr. Comp. Biol..

[54] Wang H., He X., Ji R. (2010). Selection mechanisms of *Calliptamus italicus* on four different host plants. Chin. J. Ecol..

[55] Le Gall M., Overson R., Cease A. (2019). A global review on locusts (Orthoptera: Acrididae) and their interactions with livestock grazing practices. Front. Ecol. Evol..

[56] Samset B.H., Myhre G., Forster P., Hodnebrog Ø., Andrews T., Boucher O., Faluvegi G., Fläschner D., Kasoar M., Kharin V. (2018). Weak hydrological sensitivity to temperature change over land, independent of climate forcing. npj Clim. Atmos. Sci..

[57] Sun J., Lv W., Wang S., Iler A.M., Meng F., Li B., Zhou Y., Lv J., Yuan F., Luo C. (2026). Functional group and aridity regulate impacts of climate change on plant phenology: A meta-analysis. Nat. Commun..

[58] Wang T., Miao J.-P., Sun J.-Q., Fu Y.-H. (2018). Intensified East Asian summer monsoon and associated precipitation mode shift under the 1.5 C global warming target. Adv. Clim. Change Res..

[59] Luo M., Meng F., Sa C., Duan Y., Bao Y., Liu T., De Maeyer P. (2021). Response of vegetation phenology to soil moisture dynamics in the Mongolian Plateau. Catena.

[60] Liu Q., Luo D., Wang M., Song X., Ye X., Jashenko R., Ji R. (2022). Transcriptome analysis of the response to low temperature acclimation in *Calliptamus italicus* eggs. BMC Genom..

[61] Ren J.-L., Tu X.-B., Ge J., Zhao L., Zhang Z.-H. (2016). Influence of temperature on the development, reproduction, and life table of *Calliptamus italicus* (L.) (Orthoptera: Acridoidea). J. Asia-Pac. Entomol..

[62] Riahi K., Van Vuuren D.P., Kriegler E., Edmonds J., O’neill B.C., Fujimori S., Bauer N., Calvin K., Dellink R., Fricko O. (2017). The Shared Socioeconomic Pathways and their energy, land use, and greenhouse gas emissions implications: An overview. Glob. Environ. Change.

[63] Seneviratne S.I., Zhang X., Adnan M., Badi W., Dereczynski C., Luca A.D., Ghosh S., Iskandar I., Kossin J., Lewis S. (2023). Weather and climate extreme events in a changing climate. Climate Change 2021: The Physical Science Basis: Working Group I Contribution to the Sixth Assessment Report of the Intergovernmental Panel on Climate Change.

[64] Fahrig L. (2003). Effects of habitat fragmentation on biodiversity. Annu. Rev. Ecol. Evol. Syst..

[65] Chapman J.W., Reynolds D.R., Wilson K. (2015). Long-range seasonal migration in insects: Mechanisms, evolutionary drivers and ecological consequences. Ecol. Lett..

[66] Pan R., Yan J., Xia Q., Jin X. (2024). Enhancing ecological security in Ili River valley: Comprehensive approach. Water.

[67] Bale J.S., Masters G.J., Hodkinson I.D., Awmack C., Bezemer T.M., Brown V.K., Butterfield J., Buse A., Coulson J.C., Farrar J. (2002). Herbivory in global climate change research: Direct effects of rising temperature on insect herbivores. Glob. Change Biol..

[68] Guisan A., Graham C.H., Elith J., Huettmann F., Group N.S.D.M. (2007). Sensitivity of predictive species distribution models to change in grain size. Divers. Distrib..

[69] Seo C., Thorne J.H., Hannah L., Thuiller W. (2009). Scale effects in species distribution models: Implications for conservation planning under climate change. Biol. Lett..

[70] Hengl T., Mendes de Jesus J., Heuvelink G.B., Ruiperez Gonzalez M., Kilibarda M., Blagotić A., Shangguan W., Wright M.N., Geng X., Bauer-Marschallinger B. (2017). SoilGrids250m: Global gridded soil information based on machine learning. PLoS ONE.

[71] Gallien L., Münkemüller T., Albert C.H., Boulangeat I., Thuiller W. (2010). Predicting potential distributions of invasive species: Where to go from here?. Divers. Distrib..

[72] Araújo M.B., Luoto M. (2007). The importance of biotic interactions for modelling species distributions under climate change. Glob. Ecol. Biogeogr..

[73] Dormann C.F., Schymanski S.J., Cabral J., Chuine I., Graham C., Hartig F., Kearney M., Morin X., Römermann C., Schröder B. (2012). Correlation and process in species distribution models: Bridging a dichotomy. J. Biogeogr..

[74] Guisan A., Thuiller W. (2005). Predicting species distribution: Offering more than simple habitat models. Ecol. Lett..

[75] Latchininsky A., Sword G.A., Sergeev M., Cigliano M.M., Lecoq M. (2011). Locusts and grasshoppers: Behavior, ecology, and biogeography. Psyche J. Entomol..

[76] Thuiller W., Lavorel S., Araújo M.B., Sykes M.T., Prentice I.C. (2005). Climate change threats to plant diversity in Europe. Proc. Natl. Acad. Sci. USA.

[77] Di Cola V., Broennimann O., Petitpierre B., Breiner F.T., d’Amen M., Randin C., Engler R., Pottier J., Pio D., Dubuis A. (2017). ecospat: An R package to support spatial analyses and modeling of species niches and distributions. Ecography.

[78] Popova E., Semenov S., Popov I. (2016). Assessment of possible expansion of the climatic range of Italian locust (*Calliptamus italicus* L.) in Russia in the 21st century at simulated climate changes. Russ. Meteorol. Hydrol..

[79] Peterson A.T., Soberón J., Sánchez-Cordero V. (1999). Conservatism of ecological niches in evolutionary time. Science.

[80] Wiens J.J., Ackerly D.D., Allen A.P., Anacker B.L., Buckley L.B., Cornell H.V., Damschen E.I., Jonathan Davies T., Grytnes J.A., Harrison S.P. (2010). Niche conservatism as an emerging principle in ecology and conservation biology. Ecol. Lett..

[81] Olalla-Tárraga M.Á., McInnes L., Bini L.M., Diniz-Filho J.A., Fritz S.A., Hawkins B.A., Hortal J., Orme C.D.L., Rahbek C., Rodríguez M.Á. (2011). Climatic niche conservatism and the evolutionary dynamics in species range boundaries: Global congruence across mammals and amphibians. J. Biogeogr..

[82] Wiens J.J., Graham C.H. (2005). Niche conservatism: Integrating evolution, ecology, and conservation biology. Annu. Rev. Ecol. Evol. Syst..

[83] Sunday J.M., Bates A.E., Dulvy N.K. (2012). Thermal tolerance and the global redistribution of animals. Nat. Clim. Change.

[84] Lehmann P., Ammunét T., Barton M., Battisti A., Eigenbrode S.D., Jepsen J.U., Kalinkat G., Neuvonen S., Niemelä P., Terblanche J.S. (2020). Complex responses of global insect pests to climate warming. Front. Ecol. Environ..

[85] Beck J., Böller M., Erhardt A., Schwanghart W. (2014). Spatial bias in the GBIF database and its effect on modeling species’ geographic distributions. Ecol. Inform..

[86] Yoan F., Engler J.O., Dennis R.D., Jean S., Valentine J.F. (2014). Mapping Species Distributions with MAXENT Using a Geographically Biased Sample of Presence Data: A Performance Assessment of Methods for Correcting Sampling Bias. PLoS ONE.

[87] Elith J., Leathwick J.R. (2009). Species distribution models: Ecological explanation and prediction across space and time. Annu. Rev. Ecol. Evol. Syst..

[88] Soberón J. (2007). Grinnellian and Eltonian niches and geographic distributions of species. Ecol. Lett..

[89] Merow C., Smith M.J., Silander J.A. (2013). A practical guide to MaxEnt for modeling species’ distributions: What it does, and why inputs and settings matter. Ecography.

[90] Araújo M.B., New M. (2007). Ensemble forecasting of species distributions. Trends Ecol. Evol..

[91] Wenger S.J., Olden J.D. (2012). Assessing transferability of ecological models: An underappreciated aspect of statistical validation. Methods Ecol. Evol..

