# Supplementary material for: Climate Warming May Increase the Transboundary Expansion Risk of Calliptamus italicus Between Xinjiang, China and Kazakhstan
Source: Insects. 2026 Jul 9;17(7):710. doi: 10.3390/insects17070710 (PMC13410326; doi:10.3390/insects17070710)
Supplement: Supplementary file 1 [file insects-17-00710-s001.zip › insects-4366811-supplementary.pdf]

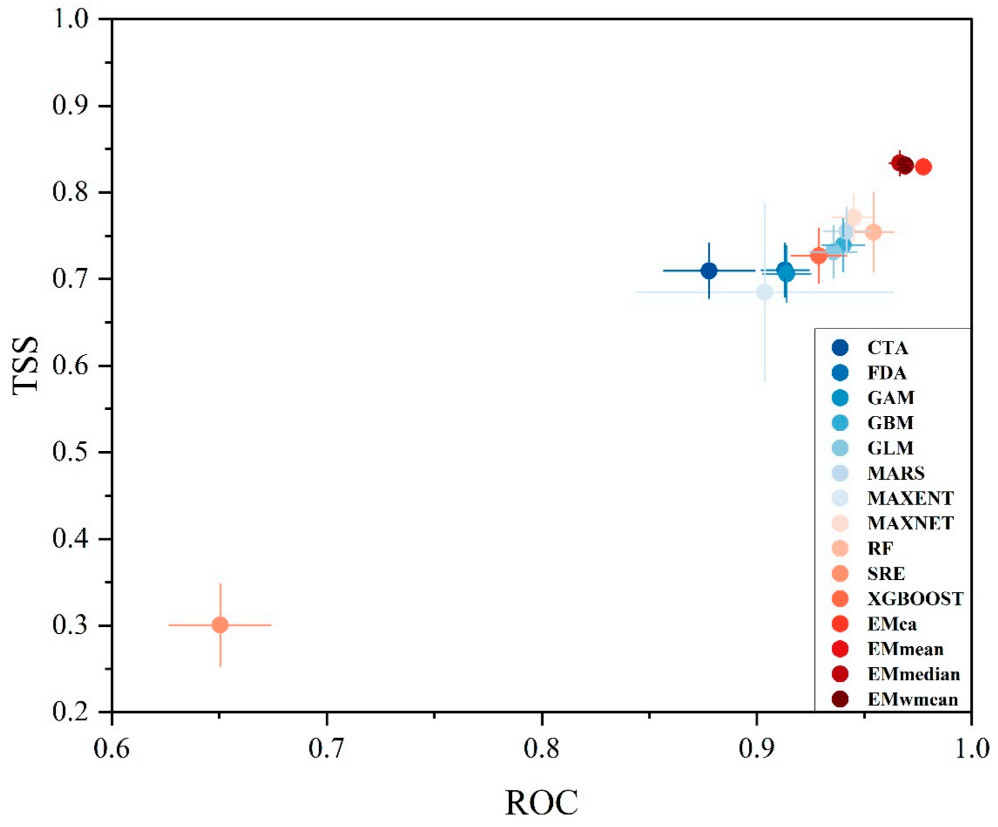

Figure S1. AUC and TSS values for single models and ensemble models

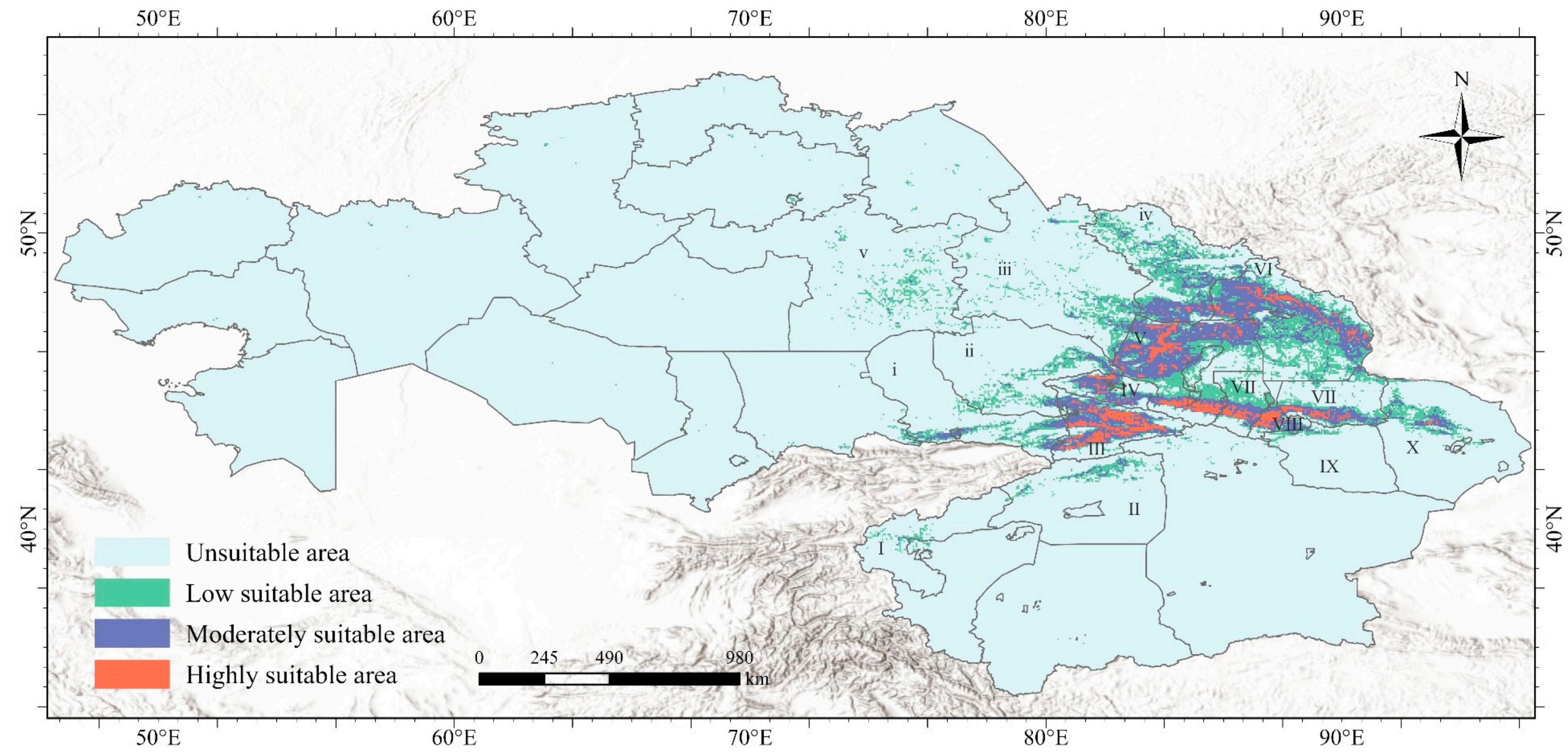

Figure S2 Potential suitable areas for *Calliptamus italicus* under current climate conditions.

Note: In Kazakhstan: i-Almaty Region; ii-Jetisu Region; iii-Abai Region; iv-East Kazakhstan Region; v-Ulytau Region.

In Xinjiang, China: I-Kizilsu Kirghiz Prefecture; II-Aksu Prefecture; III-Ili Prefecture; IV-Bortala Prefecture; V-Tacheng Prefecture; VI-Altay Prefecture;VII-Changji Prefecture; VIII-Urumqi; IX-Turpan; X-Hami.

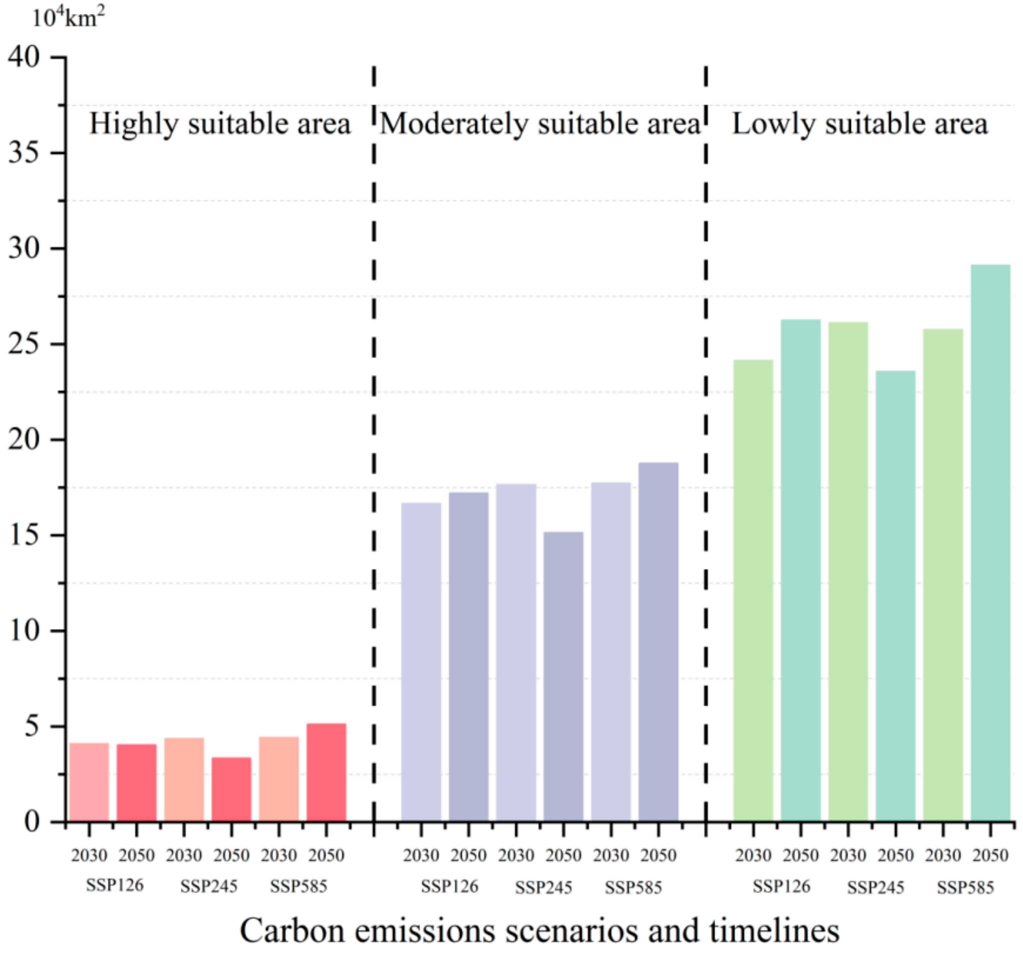

Figure S3 Area of suitable habitats under different carbon emission scenarios.

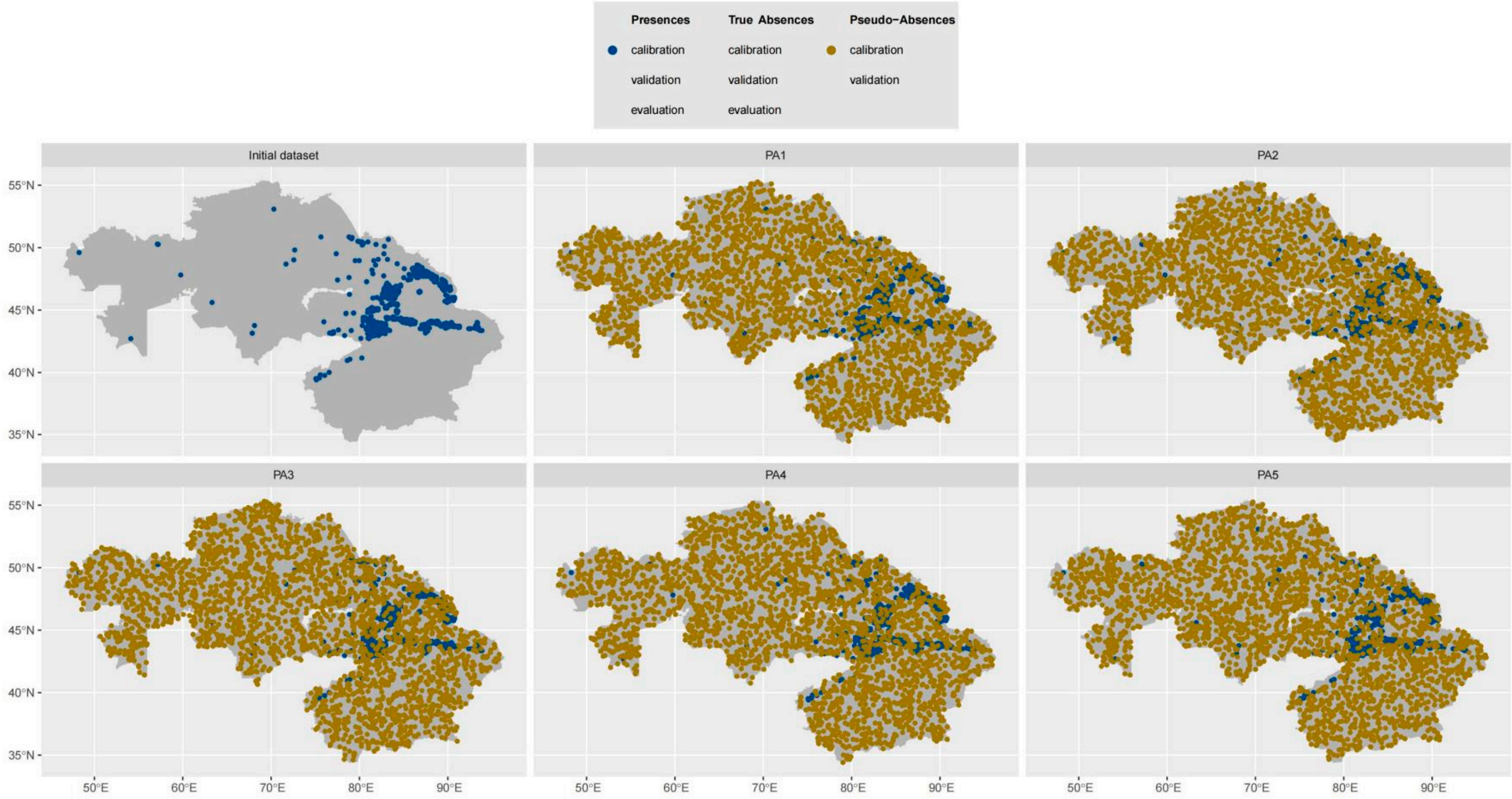

Figure S4. Distribution of pseudo-presence points and existent points.

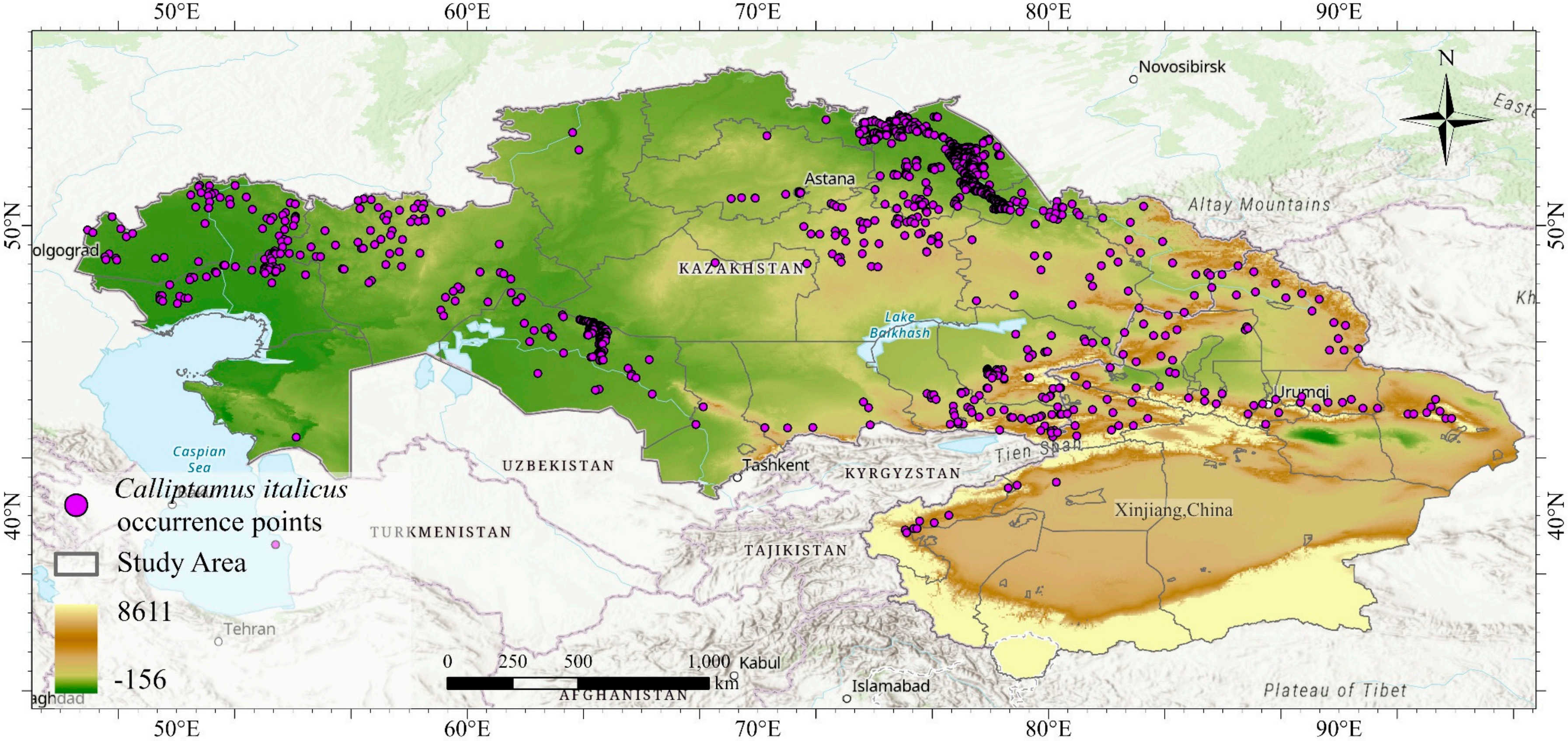

Figure S5. Distribution points of *Calliptamus italicus* including those provided by FAO CCALM.

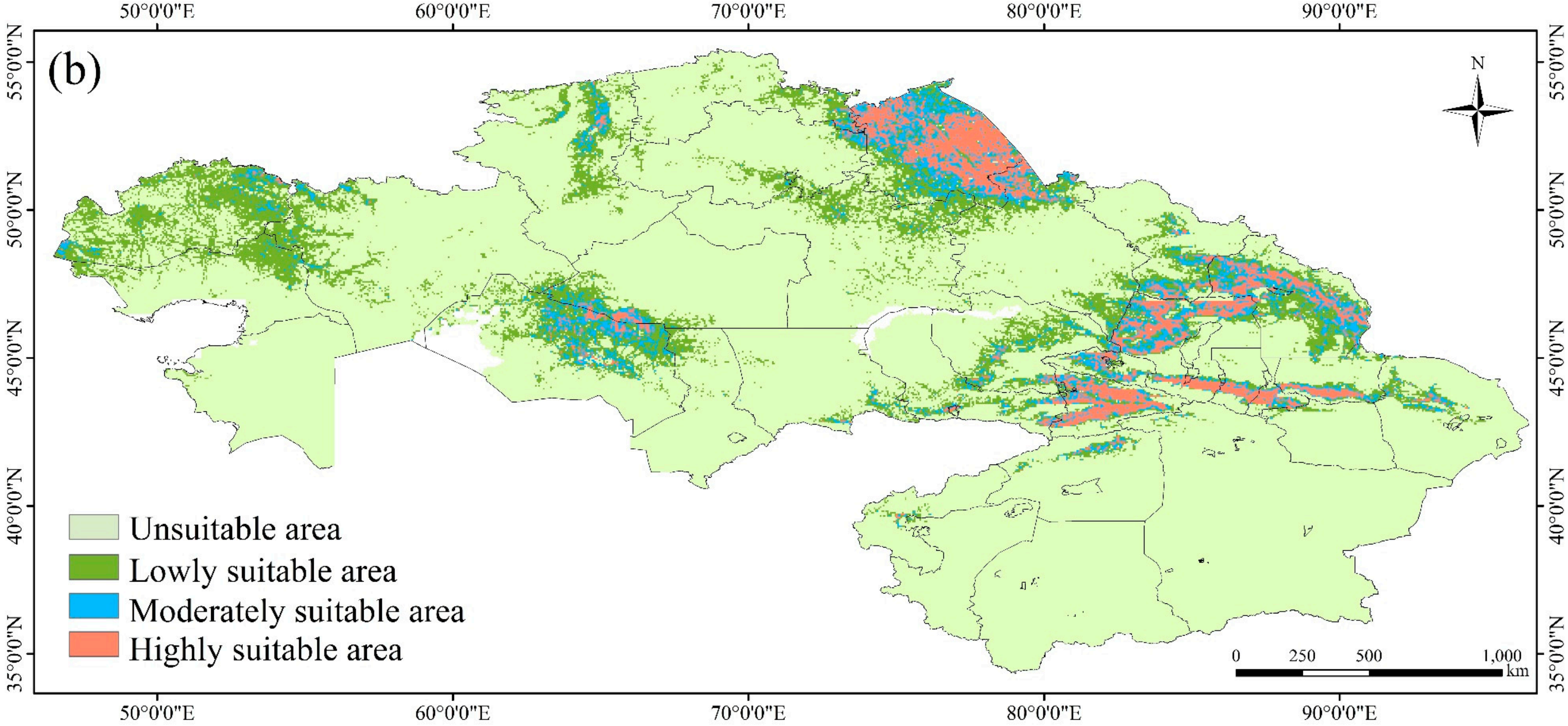

Figure S6. Potential suitable areas for *Calliptamus italicus* under current climate conditions, predicted using occurrence data including those provided by FAO CCALM.

Table S1 19 environmental factors affecting the distribution of *Calliptamus italicus*.

Types of variables	Code	Units	Description	Ecological relevance to *Calliptamus italicus*
Climatic factors	bio2	℃	Mean diurnal range	Reflects daily temperature fluctuations that may influence physiological activity, feeding behavior, and energy expenditure of individuals.
	bio4	C of V	Temperature seasonality	Represents annual temperature variability, which may affect developmental timing, diapause, and population persistence.
	bio8	℃	Mean temperature of wettest quarter	Influences growth and survival during periods when moisture conditions are relatively favorable.
	bio9	℃	Mean temperature of driest quarter	May affect egg survival and population persistence during dry seasons characteristic of arid and semi-arid habitats.
	bio10	℃	Mean temperature of warmest quarter	Closely associated with development rate, reproduction, and population growth during the active season.
	bio17	mm	Precipitation of driest quarter	Affects soil moisture conditions, egg survival, and vegetation availability during the driest period.
	bio18	mm	Precipitation of warmest quarter	Influences host plant productivity and

				habitat quality during the warmest season.
Terrain factors	elevation	m	elevation	Affects local temperature, precipitation patterns, and habitat conditions across landscapes.
	slope	°	slope	Influences drainage, soil moisture retention, and vegetation distribution.
	aspect	/	aspect	Determines solar radiation exposure and microclimatic conditions important for thermoregulation and oviposition.
Soil factors	bdod	kg/dm^3	Bulk density of the fine earth fraction	Influences soil compaction and may affect oviposition site selection and egg development.
	cec	cmol(c)/kg	Cation exchange capacity	Reflects soil fertility and nutrient availability, indirectly affecting vegetation composition and food resources.
	cfvo	cm^3/dm^3	Coarse fragments volumetric	Influences soil structure, aeration, and moisture retention, potentially affecting egg survival.
	clay	g/kg	Clay content	Affects water-holding capacity, soil texture, and suitability for oviposition.
	phh2o	/	pH in water	Influences plant community composition and soil biological processes associated with habitat quality.
	sand	g/kg	Sand content	Determines drainage and soil aeration;

				sandy soils are often preferred oviposition substrates for many grasshopper species.
	silt	g/kg	Silt content	Contributes to soil texture and moisture dynamics affecting egg incubation conditions.
	soc	g/kg	Soil organic carbon	Indicates soil productivity and may influence vegetation biomass and food availability.
Human Footprint Da ta	GHF	/	Global Human Footprint Data	Represents the intensity of human activities that may alter habitat structure, vegetation composition, and disturbance regimes.

Table S2 Mean and standard deviation of AUC and TSS values for the 11 individual algorithms across repeated model runs.

Model	AUC (mean ± SD)	TSS (mean ± SD)
CTA	0.878 ± 0.021	0.710 ± 0.032
FDA	0.913 ± 0.011	0.710 ± 0.031
GAM	0.914 ± 0.011	0.706 ± 0.032
GBM	0.940 ± 0.010	0.739 ± 0.030
GLM	0.936 ± 0.011	0.731 ± 0.030
MARS	0.942 ± 0.011	0.755 ± 0.028
MAXENT	0.904 ± 0.060	0.685 ± 0.103
MAXNET	0.945 ± 0.010	0.771 ± 0.027
RF	0.954 ± 0.009	0.744 ± 0.046
SRE	0.650 ± 0.024	0.301 ± 0.047
XGBOOST	0.929 ± 0.013	0.727 ± 0.031

Table S3 Ecological niche width and variation in principle components PC1 and PC2 between current and future projected distribution range.

Climate scenarios	Period	Levins’ B1	Levins’ B2	PC1 (%)	PC2 (%)
SSP126	2030S	0.9685	0.5252	22.41	21.23
	2050S	0.9708	0.5515	22.31	21.37
SSP245	2030S	0.9691	0.5340	22.26	21.43
	2050S	0.9678	0.5062	22.31	21.37
SSP585	2030S	0.9679	0.5206	22.5	21.16
	2050S	0.9715	0.5542	22.3	21.15

Table S4 Changes in suitable habitat area within the China–Kazakhstan border buffer under future climate scenarios

Climate scenarios	Buffer zone	Suitable habitat within border buffer ($\times 10^4 km^2$)	Change relative to current (%)
Current	100km	16.39	
2030s SSP126	100km	16.52	0.78
2030s SSP245	100km	17.15	4.64
2030s SSP585	100km	17.16	4.73
2050s SSP126	100km	16.99	3.66
2050s SSP245	100km	16.60	-4.22
2050s SSP585	100km	17.88	9.12

Table S5 Potential environmental factor affecting the distribution of *Calliptamus italicus*.

Types of variables	Code	Units	Description
Climatic factors	bio1	℃	Mean annual air temperature
	bio2	℃	Mean diurnal range
	bio3	-	Isothermality
	bio4	C of V	Temperature seasonality
	bio5	℃	Max temperature of warmest month
	bio6	℃	Min temperature of coldest month
	bio7	℃	Annual temperature range
	bio8	℃	Mean temperature of wettest quarter
	bio9	℃	Mean temperature of driest quarter
	bio10	℃	Mean temperature of warmest quarter
	bio11	℃	Mean temperature of coldest quarter
	bio12	mm	Mean annual precipitation
	bio13	mm	Precipitation of wettest month
	bio14	mm	Precipitation of the driest month
	bio15	C of V	Variation of precipitation seasonality
	bio16	mm	Precipitation of wettest quarter
	bio17	mm	Precipitation of driest quarter
	bio18	mm	Precipitation of warmest quarter
	bio19	mm	Precipitation of coldest quarter
Terrain factors	elev	m	Elevation

	slo（extracted from elev using ArcGIS software）	°	Slope
	asp（extracted from elev using ArcGIS software）	°	Aspect
Soil factors	bdod	kg/dm^3	Bulk density of the fine earth fraction
	cec	cmol(c)/kg	Cation exchange capacity
	cfvo	cm^3/dm^3	Coarse fragments volumetric
	clay	g/kg	Clay content
	phh2o	/	pH in water
	sand	g/kg	Sand content
	silt	g/kg	Silt content
	soc	g/kg	Soil organic carbon
	nitrogen	g/kg	Total Nitrogen
	ocd	kg/m^3	Organic Carbon Density
Human Footprint Data	GHF	/	Global Human Footprint Data

Table S6 Comparison of predicted suitable habitats within the 100km Xinjiang, China–Kazakhstan border buffer based on the original and CCALM-expanded occurrence datasets.

Metric	Original	Expanded
Occurrence records	559	1318
Suitable area in 100-km border buffer (cells)	7449	6630
Shared suitable area (cells)	5769	5769
Difference (%)	11.0	
Proportion of Expanded suitable area also identified by Original model (%)	87.0	
